# Further evidence in favour of a carbanion mechanism for glycolate oxidase

**DOI:** 10.1002/2211-5463.13534

**Published:** 2023-03-27

**Authors:** Hélène Pasquier, Florence Lederer

**Affiliations:** ^1^ CNRS UMR 8000, Faculté des Sciences, Institut de Chimie Physique Université Paris‐Saclay Orsay France

**Keywords:** carbanion, chemical mechanism, glycolate oxidase, lactate, trifluorolactate

## Abstract

The flavoenzyme glycolate oxidase oxidizes glycolic acid to glyoxylate and the latter, more slowly, to oxalate. It is a member of an FMN‐dependent enzyme family that oxidizes l‐2‐hydroxy acids to keto acids. There has been a controversy concerning the chemical mechanism of substrate oxidation by these enzymes. Do they proceed by hydride transfer, as observed for NAD‐dependent enzymes, or by initial formation of a carbanion that transfers the electrons to the flavin? The present work describes a comparison of the reactivity of glycolate, lactate and trifluorolactate with recombinant human glycolate oxidase, by means of rapid‐kinetics experiments in anaerobiosis. We show that trifluorolactate is a substrate for glycolate oxidase, whereas it is known as an inhibitor for NAD‐dependent enzymes, as is trifluoroethanol for NAD‐dependent alcohol dehydrogenases. Unexpectedly, it was observed that, once reduced, a flavin transfers an electron to an oxidized flavin, so that the end species is a flavin semiquinone, whatever the substrate. This phenomenon has not previously been described for a glycolate oxidase. Altogether, considering that another member of this flavoenzyme family (flavocytochrome *b*_2_, a lactate dehydrogenase) has also been shown to oxidize trifluorolactate (Lederer F et al. (2016) Biochim Biophys Acta 1864, 1215–21), this work provides another important piece of evidence which is hardly compatible with a hydride transfer mechanism for this flavoenzyme family.

## Graphical abstract

It is shown that trifluorolactate is a substrate for glycolate oxidase. Flavin reduction by trifluorolactate is a mechanistic proof for the intermediate formation of a carbanion. Surprisingly, for glycolate, lactate and trifluorolactate, the reduction end product is a flavin semiquinone. Global analysis of the flavin reduction kinetics suggests an inter‐tetramer electron transfer.

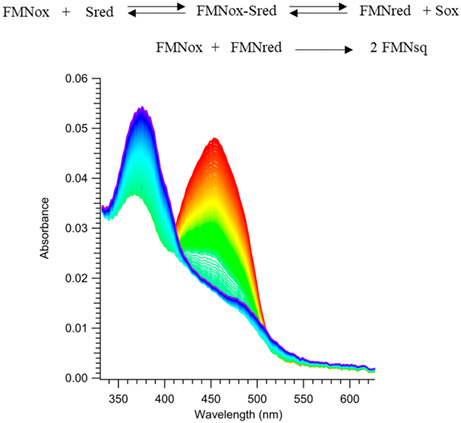

**Abbreviations**
DCIP: dichlorophenol indophenol
F3Lac: trifluorolactate
Fcb2: flavocytochrome *b*_2_
FMN: flavin mononucleotide
hGOX: human glycolate oxidase
LCHAO: long‐chain hydroxy acid oxidase
LMO: lactate monooxygenase from *M. smegmatis* (formerly called lactate oxidase)
LOX: lactate oxidase from *A. viridans*
MD: molecular dynamics
MDH: mandelate dehydrogenase
QM/MM: quantum mechanics/molecular mechanics
sGOX: spinach glycolate oxidase

The peroxisomal flavoenzyme glycolate oxidase (EC1.1.3.15, isozyme A, also called short‐chain hydroxy acid oxidase or HAOX1) is present in plants and animals. The tetrameric enzyme catalyses the oxidation of glycolate to glyoxylate at the expense of oxygen and, more slowly, of hydrated glyoxylate to oxalate [1, 2]. In plant leaves, it is involved in the photorespiratory cycle. Among plant glycolate oxidases, the spinach one (sGOX) has been the best studied at the molecular level, with kinetic characterizations of the wild‐type and variant enzymes, and determination of crystal structures with and without inhibitors [3, 4, 5, 6, 7, 8]. In humans, it is expressed primarily in the liver [9]. Glyoxylate, a toxic compound, can be detoxified to glycine by alanine‐glyoxylate transaminase (AGT) in the peroxisome; in the cytosol, it can be reduced back to glycolate by glyoxylate‐hydroxy pyruvate reductase (GR/HPR) or oxidised to oxalate by an NAD^+^‐dependent lactate dehydrogenase. Genetic deficiencies of AGT or GR/HPR lead to primary hyperoxaluria type I and type II, respectively, diseases characterized by the accumulation of calcium oxalate stones, in the kidneys in particular [10]. In mammals, the pig liver enzyme [11, 12, 13, 14] and the one from human liver as well as its recombinant form (hGOX) were characterized at the molecular level [15, 16, 17, 18, 19, 20].

Glycolate oxidase is a member of the family of FMN‐dependent l‐2‐hydroxy acid‐oxidizing enzymes. Other well‐characterized members include its isozyme B (long‐chain hydroxy acid oxidase, LCHAO or HAOX2), as well as microbial lactate oxidase (LOX) and lactate monooxygenase (LMO); dehydrogenases‐electron transferases such as flavocytochrome *b*_2_ (Fcb2, an l‐lactate cytochrome *c* oxido‐reductase) and mandelate dehydrogenases (MDH) have also been characterized. Crystal structures of these enzymes show a well‐conserved β_8_α_8_ barrel with FMN bound at its C‐terminal end [3, 4, 5, 7, 21, 22, 23, 24, 25]. Active site residues are also well conserved, with a few substitutions due to different substrate specificity. As loop 4, between β‐strand 4 and α‐helix 4, is flexible, the full loop is not always visible in crystal structures. Depending on the ligand in the active site, or even between subunits in the same crystal unit, the visible length can be different or have a different conformation [16, 17, 22, 23, 24, 25, 26, 27, 28, 29, 30, 31, 32, 33]. These structures, combined with results from solution studies [30, 31, 32, 33, 34, 35, 36, 37], show that loop 4 contributes to catalysis. A few residues in or close to the active site pocket also display mobility, such as W110 in hGOX [16] and its homologue W108 in sGOX [6] or invariant R164 in sGOX [7] and its homologue R289 in Fcb2 [38] (Fig. 1). This mobility does not facilitate understanding the side chains mechanistic role. The case of W110(108) is particularly interesting. This residue, which is unique to glycolate oxidases at this position in the family, is close to the active site. The W108S variant of sGOX had a 500‐fold lower *k*_cat_ and a strikingly higher *K*_m_ for glycolate compared to the WT [6]. It was concluded that this residue may play a role in substrate specificity. Indeed, for the spinach and the human enzyme, crystal structures of complexes with several inhibitors showed an adaptation of the side‐chain conformation which in some cases even induced movements of other residues extending to loop 4 [7, 16].

**Fig. 1:**
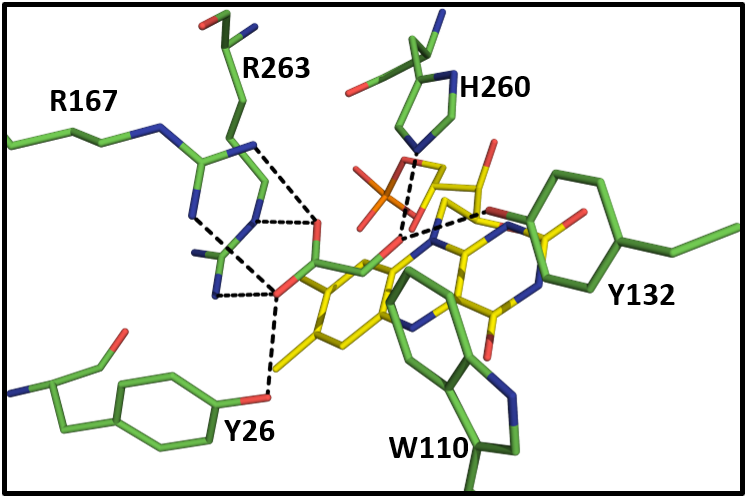
hGOX active site with bound glyoxylate, drawn from the crystal structure (Protein Data Bank 2RDU). Atom colouring: red for oxygen, blue for nitrogen, yellow for the flavin carbon atoms and green for the other amino acids and glyoxylate. The side chains in the figure are conserved in other family members, except for Y26 (phenylalanine in LCHAO) and, importantly, for W110 which is specific to GOX; in other family members, it is replaced by a variety of residues. The hydrogen bonds and other close interaction are shown by dotted lines, as described in [16]. Glyoxylate is here in its unhydrated form and can be considered as playing the role of an inhibitor. In the Fcb2 crystal structure with bound pyruvate (Protein Data Bank 1FCB), the hydrogen bonding interactions are identical to those shown here, with the exception that here mobile hGOX R167 is in the so‐called proximal position and interacts with the ligand carboxylate, while its Fcb2 homologue R289 is in the so‐called distal position (Fig. S1), interacting with D292, an invariant residue in the family [21]. In the 1FCB Fcb2 structure, the flavin is in the semiquinone state [21, 62] in agreement with the evidence that pyruvate stabilizes the flavin semiquinone [63].

Three possible chemical mechanisms were considered for the oxidation of a carbon–hydrogen bond by these enzymes, namely a hydride transfer mechanism, a proton/electron mechanism and a carbanion mechanism [39, 40]. Experimental evidence in favour of the substrate α‐hydrogen abstraction as a proton, followed by electron transfer from the carbanion to the flavin, had been proposed early on for LMO [41]. When the first crystal structures became available, the active site base was identified as a histidine (H260 in hGOX and H373 in Fcb2) (Fig. 1). Substrate modelling, starting from the pyruvate molecule orientation in Fcb2 crystals [21] suggested two possible substrate‐binding modes [42, 43]. In the first one, the C2 hydrogen was pointing towards the catalytic histidine N3, ready for abstraction as a proton, with formation of a carbanion (Fig. S1A); in the second one, obtained by a 40° rotation of the substrate C1–C2 bond, the catalytic histidine N3 was hydrogen bonded to the substrate hydroxyl group, orienting the lactate C2 hydrogen towards flavin N5 for a hydride transfer (Fig. S1B).

Numerous studies have tried to understand the role of active site residues in catalysis and to solve the mechanistic issue, using combinations of site‐directed mutagenesis, kinetic studies (with a variety of substrates and inhibitors) including solvent and pH effects, primary kinetic isotope effects as well as crystallography. MD and QM/MM computations were also carried out on a minimal model of the Fcb2 active site [44], on its whole flavodehydrogenase domain [45] and on LCHAO [46]. In some cases, experimental results led to ambiguous conclusions. But in other cases, pieces of evidence were provided that are hardly compatible or even incompatible with a hydride transfer mechanism [43, 47, 48, 49, 50, 51, 52, 53, 54, 55, 56, 57].

In this work, we analyse the reactivity of trifluorolactate (F3Lac) with hGOX, and compare it to that of lactate and glycolate. It is expected that electron attraction by the three fluorine atoms will impede the C2 hydrogen removal as a hydride ion. Indeed, F3Lac has been shown not to be a substrate but an inhibitor of NAD‐dependent lactate dehydrogenases from four different species [58, 59, 60, 61]. We recently showed that F3Lac is a substrate for Fcb2 [49]. Here, we show that F3Lac is also a substrate for glycolate oxidase, another piece of evidence pointing to a carbanion mechanism.

## Results

We have compared the reactivity of hGOX with three α‐hydroxy acids, glycolate, l‐lactate and trifluorolactate (F3Lac). The latter had never been tested with this enzyme and the present work demonstrates that it is a substrate. This has important mechanistic consequences. Moreover, for the three substrates, we report a phenomenon which had never been observed previously, namely the formation of a flavin semiquinone by an electron transfer between a reduced and an oxidized flavin during the reduction process.

### Unexpected flavin semiquinone formation after reduction

**Fig. 2:**
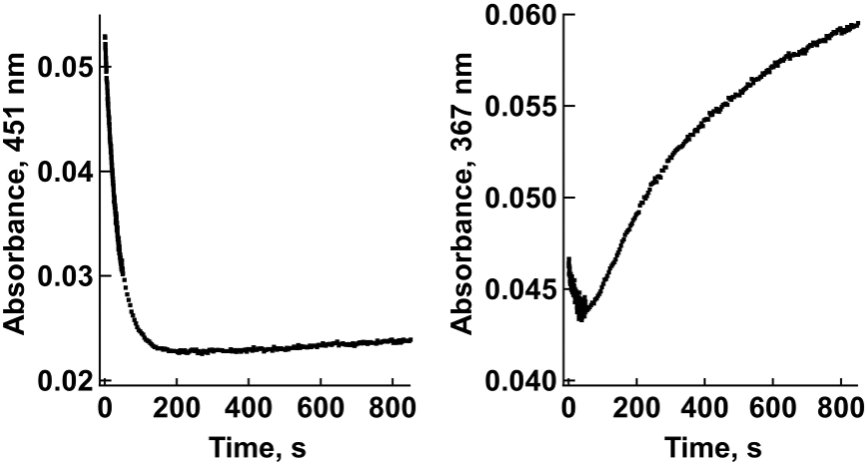
Evolution of the absorbance at 451 nm (left) and 367 nm (right) upon hGOX reduction by dl‐F3Lac (3.9 mm). Similar traces were obtained with glycolate and lactate (Figs S2 and S3). For more details, see Materials and methods.

**Fig. 3:**
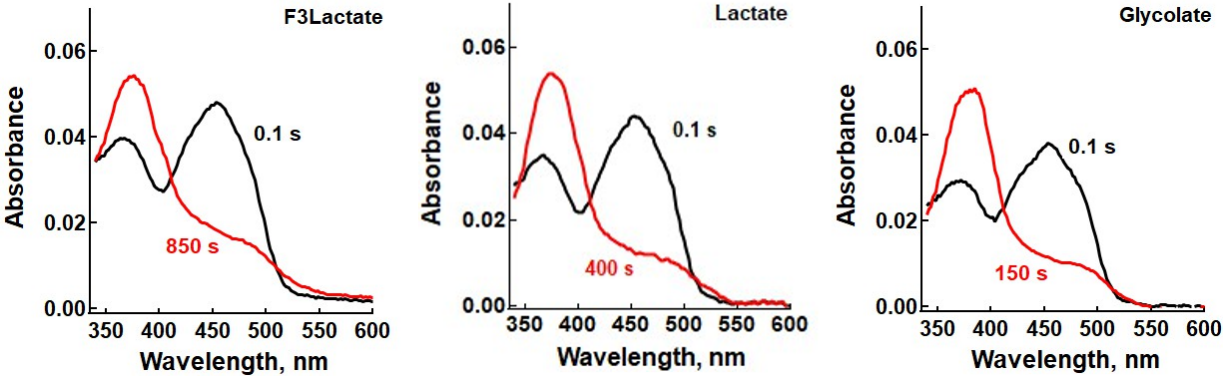
First and last spectra recorded during enzyme reduction by 3.9 mm dl‐F3Lac, 5 mm l‐lactate and 5 mm glycolate.

The time course of flavin reduction by glycolate, l‐lactate and dl‐F3Lac was monitored over several hundred seconds using a diode array stopped‐flow spectrophotometer under anaerobic conditions, as detailed in Materials and methods. For the three substrates, the initial absorption spectrum was consistent with that of the oxidized flavin form (Fig. 3). Analysis of the absorbance changes versus time at 451 and 367 nm (close to the two maxima of the oxidized flavin spectrum) pointed out at least two kinetic phases. At 451 nm, the absorbance started decreasing with time in a nearly monoexponential mode (Fig. 2, left; Figs S2 and S3 for glycolate and lactate); at longer times, from a few seconds for glycolate to a few dozen seconds for dl‐F3Lac, a second increasing phase of low amplitude appeared. At 367 nm (Fig. 2, right; Figs S2 and S3), the initial phase of decreasing absorbance corresponding to flavin reduction was followed by an absorbance increase of higher amplitude. This second phase, ascribed to formation of a flavin semiquinone, suggested a one‐electron transfer from a reduced flavin to an oxidized one. In other words, the flavin reduced form was not the final product of the reaction, but an intermediate. Altogether, the end spectrum was not that of reduced flavin, but that of the anionic flavin semiquinone (Fig. 3).

### Determination of rate constants

For determining the rate constants, in order to avoid simplifying approximations, we globally fitted the total time course of the transients from a few milliseconds to several hundred seconds using numerical data analysis. This provided the evaluation of the dependence on substrate concentration of all the rate constants involved in the formation of the flavin semiquinone. For each substrate, the spectra of the different species were averaged over the spectra derived from the global fit, regardless of the substrate concentration.

The question was whether the interflavin electron transfer was taking place within a tetramer or between tetramers. Each dataset was fitted by distinct mechanisms assuming an electron transfer either between two different tetramers or between two subunits in the same tetramer. Whatever the substrate and its concentration, for mechanisms involving an inter‐tetramers electron transfer, the quality of the fits was better than for those involving intra‐tetramer electron transfer. In the following, only the results obtained by fitting the data with the inter‐tetramers mechanism are presented and discussed. In the Supporting Information, Figs S5–S7 compare the fits assuming either an intra‐tetramer or an inter‐tetramers electron transfer for the three substrates.

For lactate and F3Lac, the inter‐tetramers mechanism A was used as the kinetic model to globally fit the datasets.

### Mechanism A

$$\begin{array}{l} \text{FMNox}+\text{Sred}\;\underset{\text{Step}\;1}{\;\overset{k_{1}}{\underset{k_{2}}{⇄}}}\;\text{FMNox‐Sred}\;\underset{\text{Step}\;2}{\;\overset{k_{3}}{\underset{k_{r3}}{⇄}}}\;\text{FMNred}+\mathrm{Sox}\\ \;\text{FMNox}+\text{FMNred}\;\overset{k_{4}}{\underset{\text{Step}\;3}{\to }}\;2\;\text{FMNsq} \end{array}$$

The reversibility of the flavin reduction step (step 2) had to be introduced in order to enable the formation of a flavin semiquinone at long times in combination with flavin reduction. Indeed, the absence of reversibility in step 2 favours the accumulation of the reduced form over time, limiting flavin semiquinone formation (Fig. S4). This reversibility is supported by the examination of the residuals which clearly showed that the model without reversibility was not appropriate and did not allow to fit the data (Fig. S5A). The introduction of reversibility in step 2 significantly decreased the value of the AIC criterion (definition in Materials and methods) and improved the quality of the global fit resulting in small, evenly distributed residuals (Fig. S5B). The introduction of reversibility is supported by published results showing that Fcb2, when reduced in anaerobiosis by lactate in excess, can reduce ketoacids such as halogeno pyruvates [48, 50] (Scheme S1). This capacity was confirmed for hGOX (unpublished experiments) and LCHAO [64]. Similarly, it was shown that reduced MDH from *Pseudomonas putida* can reduce its normal product benzoylformate [27, 55].

Mechanism A enabled an accurate description of the data obtained with F3Lac (Fig. S5) and lactate (Fig. S6). In contrast, it failed to provide good fits for glycolate (Fig. S7). In this case, the reactions of the inter‐tetramers mechanism B were used as the kinetic model for the global analysis.

### Mechanism B

$$\begin{array}{l} \text{FMNox}+\text{Sred}\;\underset{\text{Step}\;1}{\;\overset{k_{1}}{\underset{k_{2}}{⇄}}}\;\text{FMNox‐Sred}\;\underset{\text{Step}\;2}{\;\overset{k_{3}}{\underset{k_{r3}}{⇄}}}\;\text{FMNred‐Sox}\;\underset{\text{Step}\;3}{\overset{k_{5}}{\underset{k_{r5}}{⇄}}}\mathrm{FMNred}\;+\mathrm{Sox}\;\;\\ \;\text{FMNox}+\text{FMNred}\;\overset{k_{4}}{\underset{\text{Step}\;4}{\to }}\;2\;\text{FMNsq} \end{array}$$

For this substrate, an additional reversibility step had to be added for the dissociation of the FMNred‐Sox complex; this suggests that this product of the physiological substrate has a higher affinity for the active site than have the products of lactate and F3Lac oxidation. This is supported by the crystallization of an hGOX‐glyoxylate complex when the enzyme was mixed with glycolate under proper conditions [16]. The introduction of an intermediate species in the kinetic model clearly improves the residuals, and mechanism B satisfactorily matches the data (Fig. S7). The decrease in AIC value (see definition in Materials and methods), in spite of the increase in the number of parameters to be fitted, underlines the significant improvement in the quality of the fit to mechanism B for glycolate compared to mechanism A.

**Fig. 4:**
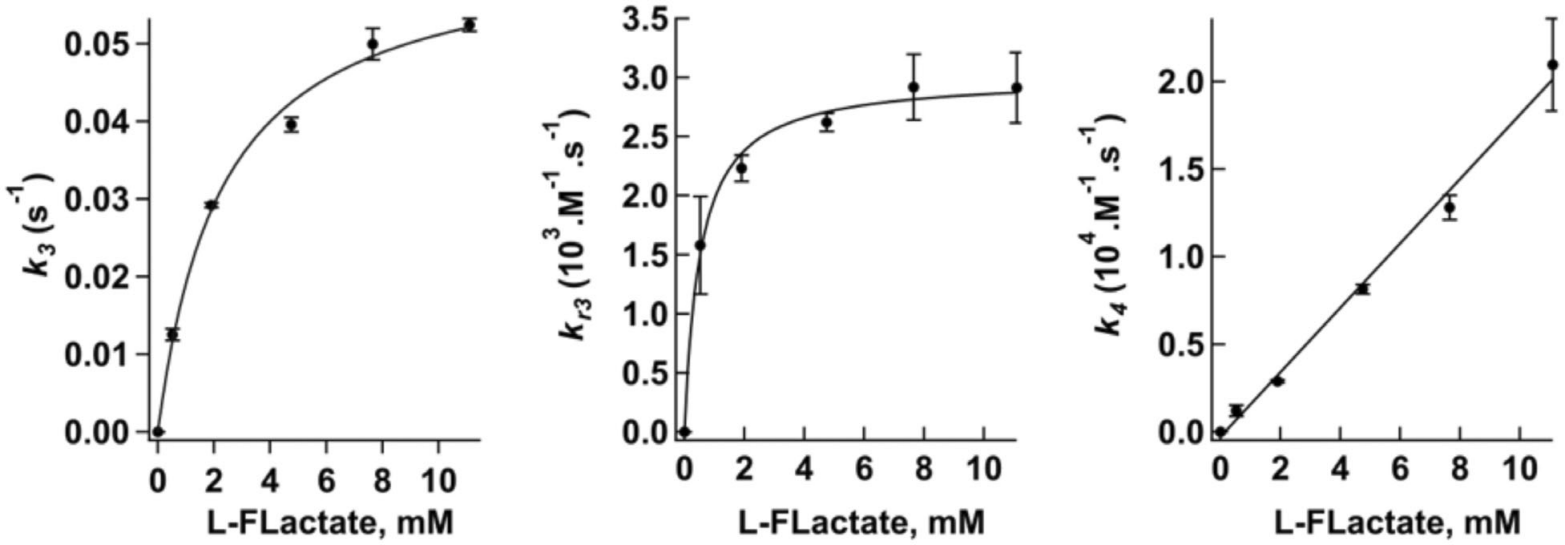
Dependence of the kinetic parameters on F3Lac concentration. The experiments were carried out with the racemic F3Lac but the results are expressed in terms of the l‐stereoisomer concentration. Error bars represent the standard deviations (SD).

Altogether, the fact that the data are better fitted with the inter‐tetramers than with the intra‐tetramer mechanism is consistent with the fact that the distance between the flavin N5 atoms in adjacent subunits is on the order of 45 Å, a distance which makes the intra‐tetramer electron transfer less likely. The dependence of the estimated kinetic parameters on substrate concentration for an electron transfer between tetramers is shown in Fig. 4 for F3Lac and in Figs S8 and S9 for glycolate and lactate respectively. Flavin reduction (*k*_3_) follows the expected saturation curve; *k*_r3_ also reaches a plateau at high substrate concentrations. The derived enzymatic parameters for flavin reduction and semiquinone formation are given in Table 1. Altogether, F3Lac is about 10‐fold slower than lactate, and 3‐ to 4‐fold less efficient. But lactate itself is a 30‐fold slower reductant than glycolate, and is 1.4 × 10^3^‐fold less efficient. The rate of FMNsq formation (*k*_4_) increases linearly with substrate concentration and is 10‐fold slower for F3Lac than for lactate. This parameter appears to depend essentially on the rate of FMNred formation.

**Table 1:** Kinetic parameters for flavin reduction and semiquinone formation by glycolate, l‐lactate and F3Lac. The values given here were calculated using the rates obtained by computer simulation of the experimental traces at the various substrate concentrations, as shown in Fig. 4 and Figs S8 and S9.

Substrate	*k*_3_		*k*_r3_		*k*_4_	*k*_5_		*k*_r5_	
	*k*_max_ (s^−1^)	*K*_1/2_ (mm)	*k*_max_ (s^−1^)	*K*_1/2_ (mm)	Slope (s^−1^·mm ^−2^)	*k*_max_ (s^−1^)	*K*_1/2_ (mm)	*k*_max_ (s^−1^)	*K*_1/2_ (mm)
Glycolate	25.05 ± 0.45	0.195 ± 0.020	6.83 ± 0.50	1.04 ± 0.22	654 × 10^6^ ± 64 × 10^6^	0.11 ± 0.02	0.67 ± 0.42	22 270 ± 204	ND
Lactate	0.78 ± 0.05	8.35 ± 1.44	5037 ± 388	1.99 ± 0.55	18.0 × 10^6^ ± 2.4 × 10^6^				
F3Lac^a^	0.063 ± 0.003	2.29 ± 0.03	3008 ± 80	0.53 ± 0.08	1.84 × 10^6^ ± 0.08 × 10^6^				

^a^ Results expressed in terms of the l‐stereoisomer.

**Fig. 5:**
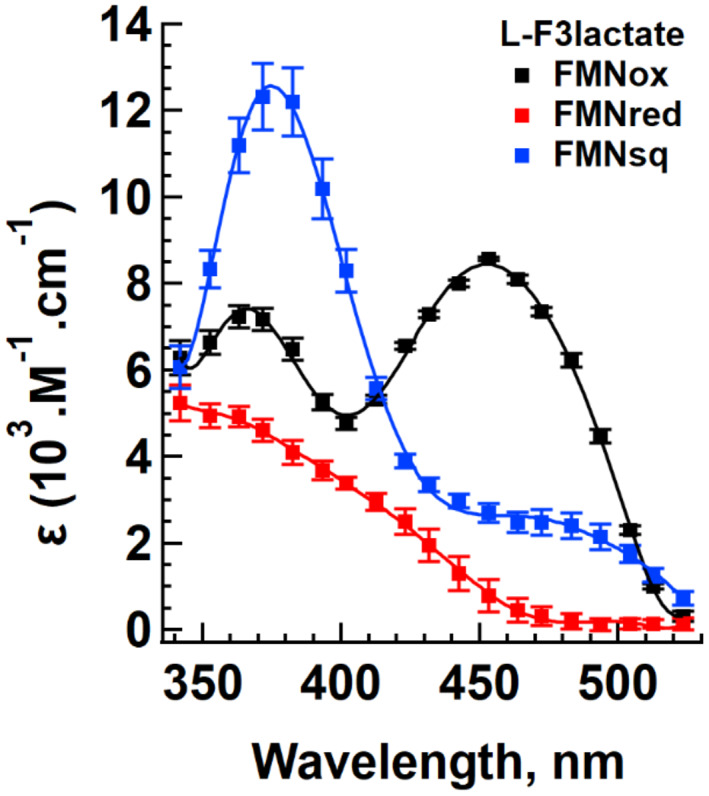
Reaction with dl‐F3lactate: calculated spectra of the three redox states resulting from the global analysis of the experimental data. For the reactions with glycolate and lactate, the spectra are given in Figs S10 and S11. Error bars represent the standard deviations (SD).

The global fit allowed to reconstruct the shape of the absorption spectra of the three species involved (Fig. 5; Figs S10 and S11). It was checked that the shape of these spectra did not depend significantly on substrate concentration. Within experimental error, they correspond satisfactorily to the experimental ones, as well as to those reported in the literature. In particular, the reconstituted flavin semiquinone spectrum shows the same slight red shift in the 360–370 nm region compared to the low‐wavelength maximum of the oxidized species; it also has the shoulder in the 480–500 nm region, typical of the flavin anionic semiquinone spectrum (Fig. 3). This spectrum is similar to that of sGOX produced by photoreduction [65] and of the pig liver GOX produced by coulometric titration [14].

## Discussion

By characterizing the reactivity of F3Lac as a substrate for GOX, this work provides important mechanistic information concerning the oxidation of l‐2‐hydroxy acids by glycolate oxidase, information which can be extended to other members of its family, as will be discussed later. But this work also describes the unexpected formation of a flavin semiquinone, which will be discussed first.

### Flavin semiquinone formation

Under our reaction conditions (anaerobiosis at pH 7 and 30 °C), the semiquinone was formed by electron transfer between a reduced flavin and an oxidized one, whatever the substrate. This phenomenon has never been reported for the physiological substrate glycolate. It was not observed with the spinach enzyme, at pH 8.3 and 4 °C after 10 min in a diode array spectrophotometer [65]. Using a 100 mm phosphate buffer but at pH 7 and 30 °C as in the present work, Pennati and Gadda [19] did not identify the phenomenon when monitoring hGOX reduction over 1 s at 450 nm. The existence of a slow phase of small amplitude at the end of their observation time can possibly be interpreted as indicating incipient semiquinone formation.

In itself, the phenomenon suggests that the redox potential values of the E.FMN_red_/E.FMN_sq_ and E.FMN_sq_/E.FMN_ox_ couples are close. For the pig liver enzyme at pH 7.1 and 10 °C in 100 mm phosphate buffer, they were determined to be – 30 mV and −17 mV respectively [14]. There is about 92% sequence identity between the pig liver and the human enzyme; the crystal structure of the latter (Protein Data Bank 2NZL for ex.) indicates that all the substitutions are located on the surface, except one which is not close to the flavin. Thus, one may assume that the human enzyme has similar redox potentials at the same pH in our 50 mm phosphate buffer at 30 °C.

### About the chemical mechanism of substrate oxidation

Our experimental results show that replacing the lactate CH_3_ group by the electron attracting CF_3_ group does not prevent F3Lac from being a substrate for glycolate oxidase. This is in stark contrast with the fact that F3Lac is an inhibitor for several NAD‐dependent lactate dehydrogenases [58, 59, 60, 61]. Similarly, trifluoroethanol is known as an inhibitor of NAD‐dependent alcohol dehydrogenases [66, 67, 68, 69]. Numerous studies with these enzymes from horse liver and yeast have used this compound in solution as a ligand and inhibitor [70, 71, 72, 73]. Another example of the negative effect of the CF_3_ group in a hydride transfer mechanism is that of trifluoroalanine, which was co‐crystallized with d‐amino acid oxidase as an inhibitor, while alanine is a substrate for this enzyme [74, 75]. In contrast, the facilitating effect of the fluorines in the formation of a carbanion is illustrated by the case of mandelate racemase. For this enzyme, lactate is an inhibitor, while F3Lac is a substrate and can be racemized [76, 77].

In view of this facilitating effect of the fluorine atoms in a carbanion mechanism, a faster rate and a higher catalytic efficiency for F3Lac compared to lactate could have been expected (Table 1). In our previous work with Fcb2 for the same lactate/F3Lac comparison [49], the difference between the two substrates was much larger (about four orders of magnitude for the rate). But in that case, the F3Lac catalytic efficiency was still higher than that of mandelate, a very poor Fcb2 substrate [43, 49]. The F3Lac low rate and efficiency can possibly arise from several factors: a steric problem due to the replacement of the methyl group by the trifluoromethyl group (lactate itself is a poor substrate compared to glycolate, Table 1), a possible distortion of substrate binding or transition state geometry due to potential halogen bonds and, importantly, a higher redox potential of the F3Lac/F3Pyr couple compared to the lactate/pyruvate couple. The Lac/Pyr couple redox potential is −190 mV, that of the glycolate/glyoxylate couple is −87 mV at pH 7 [78] and that of the F3Lac/F3Pyr couple is on the order of −60 mV [49]. Thus, the redox potential differences between hGOX (*E*_m_ = −68 mV for the pig liver enzyme [14]) and those substrates suggest that the driving force for reduction by F3Lac is less than that for lactate. This may counteract the favourable effect of the fluorines on C2 proton abstraction. Another important but unpredictable factor is the role of side chains mobility, in particular that of W110, and of loop 4, as observed in several crystal structures [7, 16, 17] (see Introduction).

The literature provides several other pieces of evidence in favour of the carbanion mechanism for this enzyme family, evidence that cannot be rationalized by a hydride transfer mechanism [79]. One example is the formation by LMO of a catalytically competent covalent intermediate between FMN and glycolate [53, 54], formed by attack of a carbanion on the electron‐deficient oxidized flavin N5. No catalytic adduct has been detected with other family members; it could be that the formation of covalent intermediates during the oxidation of normal substrates is sterically difficult. With MDH, another important piece of evidence (among others) in favour of a carbanion mechanism is the formation, on the way to flavin reduction by the substrate at low temperature, of a transient spectral intermediate formed between an electron‐rich donor (such as a carbanion) and electrophilic FMNox [55].

Another example is the different reactivity of mandelate for enzymes of the family. Modelling studies on the Fcb2 active site of its very slow substrate mandelate suggest that it can only bind as for a hydride mechanism due to steric interference, in particular by A198 (hGOX A81) and L230 (hGOX W110) [42, 43, 80]. Thus, when both lactate and mandelate appear to bind well to Fcb2 for hydride transfer, why is mandelate such a poor substrate for this enzyme? Variants with smaller residues at these positions and at homologous ones in family members increase the mandelate oxidation rate significantly [31, 51, 81, 82, 83]. Moreover, *bona fide* mandelate dehydrogenases have smaller residues at these positions and increasing their size decreases the activity for mandelate [84, 85].

Further evidence in favour of a carbanion mechanism is provided by the dehydrohalogenation reaction of β‐halogenated substrates catalysed by several family members (LMO, Fcb2 and LCHAO) [41, 64, 86]. This reaction does not in itself constitute an absolute proof. Indeed, it was suggested that halogen elimination could occur after enzyme reduction, when a hydride would displace the halogen from the normal keto acid product, as has been shown for d‐amino acid oxidase [87]. However, a number of studies on Fcb2 WT and variant forms provided significant mechanistic information. These studies, carried out under transhydrogenation conditions (details in Scheme S1) between, for example, lactate and bromopyruvate, showed an isotope transfer from 2‐(^2^H)‐lactate or 2‐(^3^H)‐lactate to the products bromolactate and pyruvate [42, 50, 57, 88]. An inverse deuterium isotope effect was determined for bromide elimination by the WT enzyme and several variant forms [42, 50, 56, 57]. This can only result from an intermediate carbanion partitioning between isotope‐insensitive elimination and isotope‐sensitive protonation (Scheme S1). This inverse isotope effect is totally incompatible with a bromide displacement by a hydride from reduced flavin N5H.

Still more evidence is provided by the different reactivity of β‐acetylenic substrates [89, 90, 91, 92, 93, 94] or nitroethane [95, 96, 97] between GOX family members and d‐amino acid oxidase, which works by hydride transfer [98, 99].

In spite of all the evidence briefly summarized here (more evidence is discussed in [79]), hydride transfer was proposed in recent years on the basis of kinetic isotope effects on Fcb2 variant forms [100, 101], of the interpretation of crystal structures [83, 100, 102] and of the results of QM and QMM studies on Fcb2 [44, 45] and on LCHAO [46]. A critical analysis of the validity of the interpretations in several of these papers can be found in [22, 49, 52, 79].

Altogether, after the demonstration brought by the present results added to those obtained on Fcb2 with F3Lac [49], plus all the evidence briefly recalled above for other members of the family, can one still object to a carbanion mechanism for the FMN‐dependent enzymes that oxidize l‐2‐hydroxy acids?

## Materials and methods

### Materials

dl‐Trifluorolactate (dl‐F3Lac) was obtained by chemical reduction of commercial ethyl‐trifluoropyruvate with NaBH_4_, followed by hydrolysis of the ethyl ester [103]. The compound purity and structure were analysed by gas chromatography, mass spectrometry and nuclear magnetic resonance. The concentration of stock solutions was determined on an HPLC cation exchange column (AMINEX HPX 87H; BioRad, Hercules, CA, USA) developed at 30 °C with 5 mm sulfuric acid at 0.3 mL·min^−1^. The elution profile was monitored at 210 nm. The same system was used for identifying the oxidation product, trifluoropyruvic acid, by comparison with a commercial sample. The l‐lactate lithium salt, trifluoropyruvic acid, protocatechuate dioxygenase and protocatechuic acid were purchased from Sigma (St. Louis, MO, USA). All other chemicals were of analytical grade. Recombinant hGOX was expressed and purified as described [15]. It was used without cleavage of the His‐tag at the N terminus.

### Methods

The enzyme concentration was determined in terms of its flavin content (ε_452_ = 8.6 mm ^−1^·cm^−1^) [15]. The working buffer was 50 mm Na^+^/K^+^ phosphate buffer pH 7. For experiments in anaerobiosis, buffers and substrate solutions were purged by bubbling with argon from which oxygen had been scrubbed by an Alltech Big Oxygen Trap. The concentrated enzyme was separately ventilated without bubbling, and finally, diluted with the relevant deaerated buffer. A small volume (~ 1%) of 40 mm protocatechuate was added to the solutions during deaeration. Before introducing enzyme and reagents into the stopped‐flow spectrophotometer, a small volume (~ 1%) of protocatechuate dioxygenase (6.10^−3^ units·mL^−1^) was added to the solutions, as proposed by Patil and Ballou [104]. After mixing with substrate, the enzyme concentration was on the order of 5–6 μm. The absorption spectra and their evolution over time were followed with an Applied Photophysics SX20 (Applied Photophysics Limited, Leatherhead, UK) stopped‐flow spectrophotometer equipped with a diode array detector.

### Global fit analysis

Prior to the analysis of the selected absorption spectra, a baseline correction was performed in order to eliminate the instrumental drift over time. The kinetics of absorbance evolution over time were analysed at 19 wavelengths between 340 and 520 nm, at intervals of 10 nm. Each dataset consisting of these 19 series of traces was globally fitted according to mechanisms A, B, IntraA and IntraB. The associated differential rate laws are given in the Supporting Information. The global analysis was performed using mathematica software (Version 13.0, 2021; Wolfram Research, Inc., Champaign, IL, USA). The MultiNonlinearModelFit function was used to fit the data by numerically solving the differential equations and sharing the different wavelength‐independent rate constant parameters, while the molar extinction coefficients of the different species were estimated for each wavelength. The initial values of all parameters were chosen arbitrarily, except for the rate constants *k*_1_ and *k*_2_ associated with step 1 of the FMNox‐Sred complex formation (see equations in Results and, in Supporting Information, Global fit analysis and equations section). They were set to *k*_1_ = 30.10^6^ m ^−1^·s^−1^ and *k*_2_ = 450 s^−1^ for glycolate and *k*_1_ = 156.10^3^ m ^−1^·s^−1^ and *k*_2_ = 200 s^−1^ for lactate and dl‐F3Lac. The substrate concentration was introduced as an initial parameter. We assumed that the FMNox and FMNox‐Sred species exhibited the same absorption spectrum. The quality of the fit was judged by the visual inspection of the plots of residuals. Furthermore, in order to evaluate the suitability of the different kinetic models in describing the data and to identify the one that led to the best compromise between the quality of the fit and the number of parameters to be fitted, we used the Akaike information criterion (AIC). As the number of degrees of freedom (i.e. the number of parameters) are different between the kinetic models, AIC allows to compare the models and to estimate the relevance of the improvement of the fit following an increase in the number of parameters. The value of AIC was estimated according to the following equation

$$\mathrm{AIC}=N\cdot \ln\left(\frac{\mathrm{SSQ}}{N}\right)+2\cdot \left(p+1\right),$$

where *N* designates the number of data points, SSQ the sum of squares and p the number of parameters to be fitted. For each dataset, the best kinetic model chosen will be the one with the lowest AIC value.

For each substrate concentration, all rate constants presented in this work are an average value of the rate constants estimated from the global analysis of at least three datasets.

## Conflict of interest

The authors declare no conflict of interest.

## Author contributions

FL conceived the work and carried out the experiments; HP proposed the kinetic models and carried out data analysis; FL and HP contributed to manuscript writing.

## Supporting information


**Fig. S1.** Substrate modelling from the pyruvate coordinates in the Fcb2 crystal structure 1FCB.
**Fig. S2.** Evolution of the absorbance at 451 nm and 367 nm upon hGOX reduction by 5 mM glycolate over 0.5 s and 1000 s.
**Fig. S3.** Evolution of the absorbance at 451 nm (left) and 367 nm (right) upon hGOX reduction by 5 mM L‐lactate.
**Fig. S4.** Simulated time evolution of concentrations of FMNred and FMNsq as a function of kr3 (Mechanism A), for reduction with L‐F3Lac.
**Fig. S5.** Global numerical analysis of the absorbance variation overtime at 19 wavelengths from 340 to 520 nm at intervals of 10 nm, upon hGOX reduction by 3.9 mM DL‐F3Lac.
**Fig. S6.** Global numerical analysis of the absorbance variation overtime at 19 wavelengths from 340 to 520 nm, at intervals of 10 nm upon hGOX reduction by 10 mM L‐Lactate.
**Fig. S7.** Global numerical analysis of the absorbance variation overtime at 19 wavelengths from 340 to 520 nm, at intervals of 10 nm upon hGOX reduction by 5 mM glycolate.
**Fig. S8.** Dependence of the kinetic parameters on glycolate concentration.
**Fig. S9.** Dependence of the kinetic parameters on lactate concentration.
**Fig. S10.** Reaction with glycolate: spectra of the three redox states resulting from the global analysis of the experimental data.
**Fig. S11.** Reaction with lactate: spectra of the three redox states resulting from the global analysis of the experimental data.
**Scheme S1.** Principle of the transhydrogenation reaction.
**Data S1.** Global fit analysis and equations.
**Data S2.** Codes of PDB crystal structures of ternary complexes of alcohol dehydrogenases with NAD^+^ and trifluoroethanol.

## Data Availability

Figure 1: drawn from the Protein Data Bank (PDB) structure, code HAOX1_human, Q9UJM8 and from https://doi.org/10.1021/bi701710r. The structures corresponding to the codes given at the end of the Supporting Information are all found in the PDB. The rest of the experimental data (individual stopped‐flow recordings) are available on request from the authors.
