# Supplementary material for: Further evidence in favour of a carbanion mechanism for glycolate oxidase
Source: FEBS Open Bio. 2023 Mar 27;13(5):938–50. doi: 10.1002/2211-5463.13534 (PMC10153336; doi:10.1002/2211-5463.13534)
Supplement: Supplementary file 1 — Fig. S1. Substrate modelling from the pyruvate coordinates in the Fcb2 crystal structure 1FCB. Fig. S2. Evolution of the absorbance at 451 nm and 367 nm upon hGOX reduction by 5 mM glycolate over 0.5 s and 1000 s. Fig. S3. Evolution of the absorbance at 451 nm (left) and 367 nm (right) upon hGOX reduction by 5 mM L‐lactate. Fig. S4. Simulated time evolution of concentrations of FMNred and FMNsq as a function of kr3 (Mechanism A), for reduction with L‐F3Lac. Fig. S5. Global numerical analysis of the absorbance variation overtime at 19 wavelengths from 340 to 520 nm at intervals of 10 nm, upon hGOX reduction by 3.9 mM DL‐F3Lac. Fig. S6. Global numerical analysis of the absorbance variation overtime at 19 wavelengths from 340 to 520 nm, at intervals of 10 nm upon hGOX reduction by 10 mM L‐Lactate. Fig. S7. Global numerical analysis of the absorbance variation overtime at 19 wavelengths from 340 to 520 nm, at intervals of 10 nm upon hGOX reduction by 5 mM glycolate. Fig. S8. Dependence of the kinetic parameters on glycolate concentration. Fig. S9. Dependence of the kinetic parameters on lactate concentration. Fig. S10. Reaction with glycolate: spectra of the three redox states resulting from the global analysis of the experimental data. Fig. S11. Reaction with lactate: spectra of the three redox states resulting from the global analysis of the experimental data. Scheme S1. Principle of the transhydrogenation reaction. Data S1. Global fit analysis and equations. Data S2. Codes of PDB crystal structures of ternary complexes of alcohol dehydrogenases with NAD+ and trifluoroethanol. [file FEB4-13-938-s001.pdf]

Supporting Information

Further evidence in favour of a carbanion mechanism for glycolate oxidase

Hélène Pasquier,† and Florence Lederer †*

Institut de Chimie Physique, CNRS UMR 8000,

Université Paris-Saclay, Faculté des Sciences, Orsay, France.

*To whom to address correspondence: florence.lederer@universite-paris-saclay.fr

Content

Figure S1. Substrate modelling from the pyruvate coordinates in the Fcb2 crystal structure 1FCB.
Figure S2. Evolution of the absorbance at 451 nm and 367 nm upon hGOX reduction by 5 mM glycolate over 0.5 s and 1000 s.
Figure S3. Evolution of the absorbance at 451 nm (left) and 367 nm (right) upon hGOX reduction by 5 mM L-lactate.
Figure S4: Simulated time evolution of concentrations of FMNred and FMNsq as a function of kr3 (Mechanism A), for reduction with L-F3Lac.
Figure S5: Global numerical analysis of the absorbance variation over time at 19 wavelengths from 340 to 520 nm at intervals of 10 nm, upon hGOX reduction by 3.9 mM DL-F3Lac.
Figure S6: Global numerical analysis of the absorbance variation over time at 19 wavelengths from 340 to 520 nm, at intervals of 10 nm upon hGOX reduction by 10 mM L-Lactate.
Figure S7: Global numerical analysis of the absorbance variation over time at 19 wavelengths from 340 to 520 nm, at intervals of 10 nm upon hGOX reduction by 5 mM glycolate.
Figure S8. Dependence of the kinetic parameters on glycolate concentration.
Figure S9. Dependence of the kinetic parameters on lactate concentration.
Figure S10. Reaction with glycolate: spectra of the three redox states resulting from the global analysis of the experimental data.
Figure S11. Reaction with lactate: spectra of the three redox states resulting from the global analysis of the experimental data.
Global fit analysis and equations.
Scheme S1: Principle of the transhydrogenation reaction.
Codes of PDB crystal structures of ternary complexes of alcohol dehydrogenases with NAD^+ and trifluoroethanol.

Figure S1. Substrate modelling from the pyruvate coordinates in the Fcb2 crystal structure 1FCB. Atom coloring: red for oxygen, blue for nitrogen, yellow for carbon atoms of flavin and side chains, green for lactate. **A**: lactate orientation for a carbanion mechanism; **B:** orientation for a hydride transfer reaction. Black dotted lines indicate hydrogen bonds. Red dotted lines represent the direction of substrate α-hydrogen transfer as a proton to the active site histidine in the carbanion mechanism (**A**), as a hydride to flavin N5 (**B**). R289 is here in the distal orientation, pointing towards D292, which is not shown in the figures.

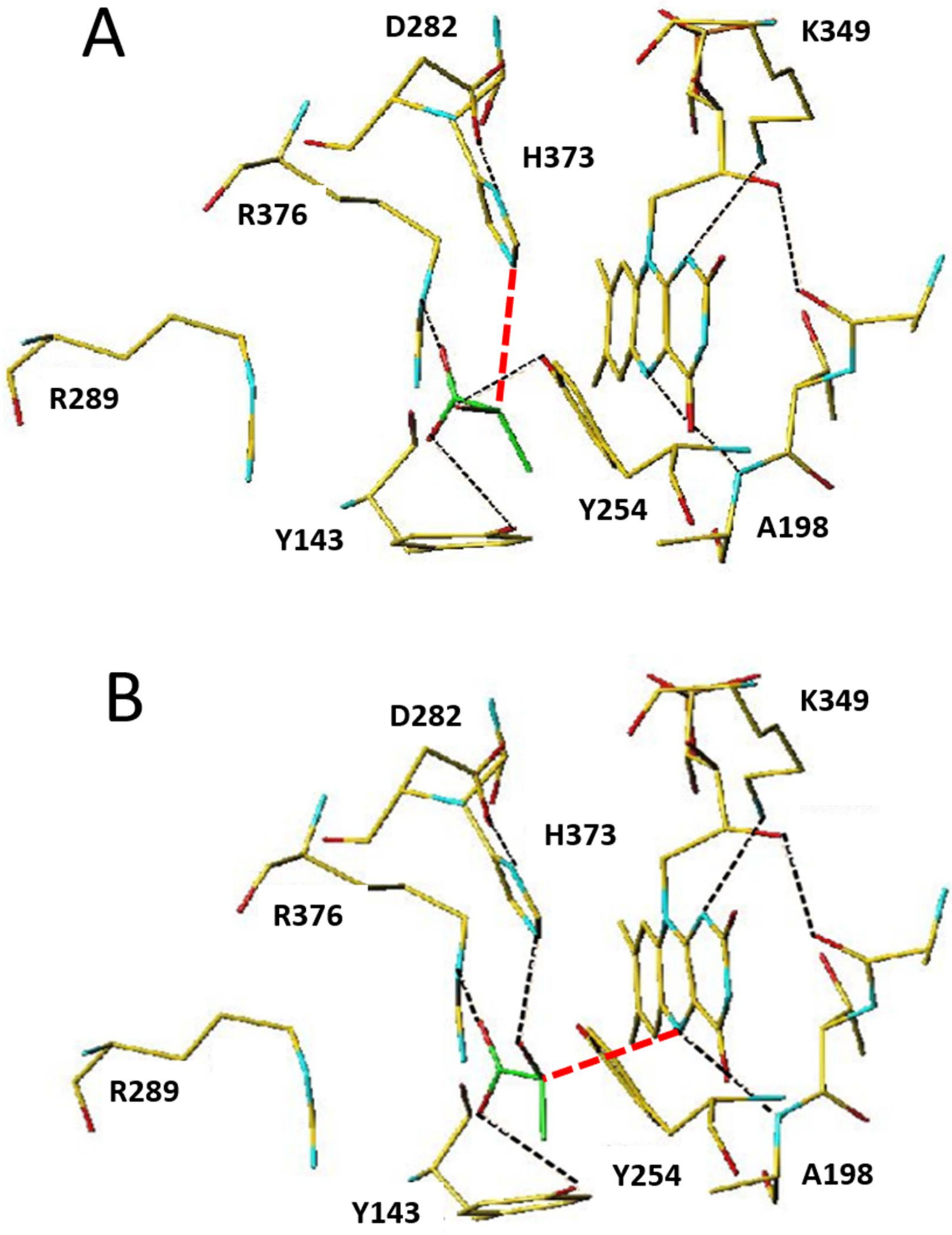

Figure S2. Evolution of the absorbance at 451 nm and 367 nm upon hGOX reduction by 5 mM **glycolate** over 0.5 s and 1000 s. For more details, see Materials and Methods.

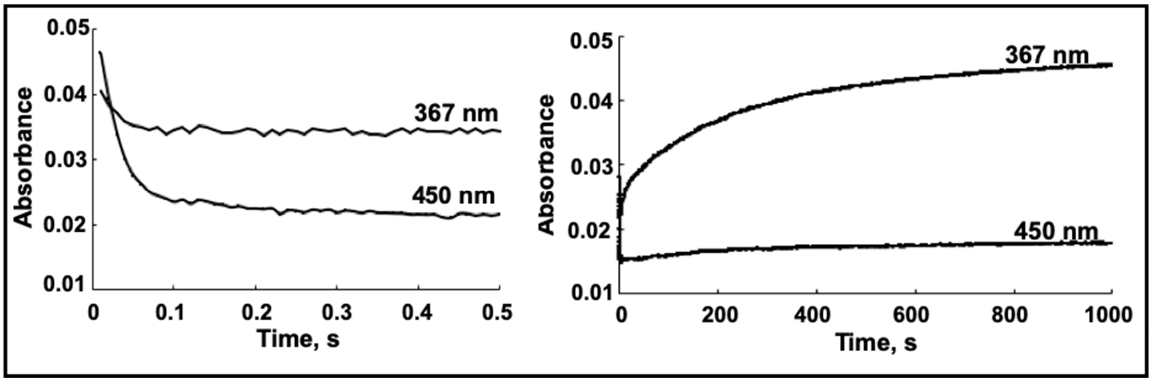

Figure S3. Evolution of the absorbance at 451 nm (left) and 367 nm (right) upon hGOX reduction by 5 mM **L-lactate**. For more details, see Materials and Methods.

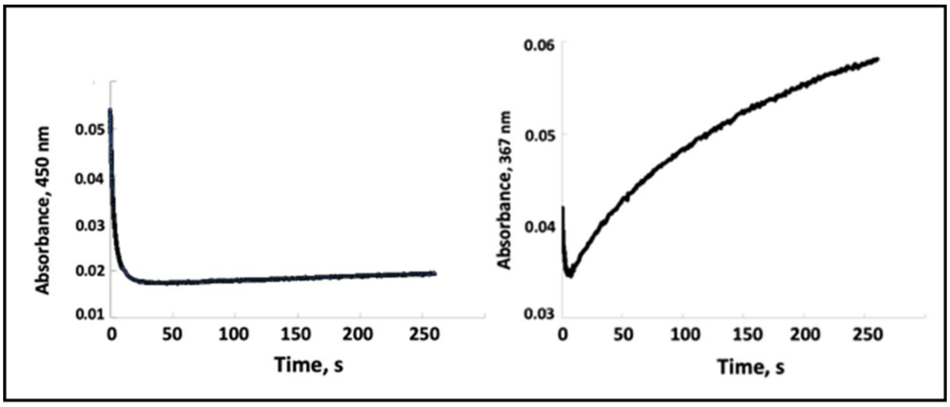

Figure S4: Simulated time evolution of concentrations of FMNred and FMNsq as a function of kr3 (Mechanism A), for reduction with **L-F3Lac**. In case of an irreversible flavin reduction (*k*r3=0 $L.mol^{-1}.s^{-1}$), semiquinone formation in the long run is limited and the system favours the accumulation of the reduced form. Here, E_0, S_0, *k*3 and *k*4 were set to 5.9 μM, 2.5mM, 0.023 s^{-1} and 4600 $L.mol^{-1}.s^{-1}$, respectively.

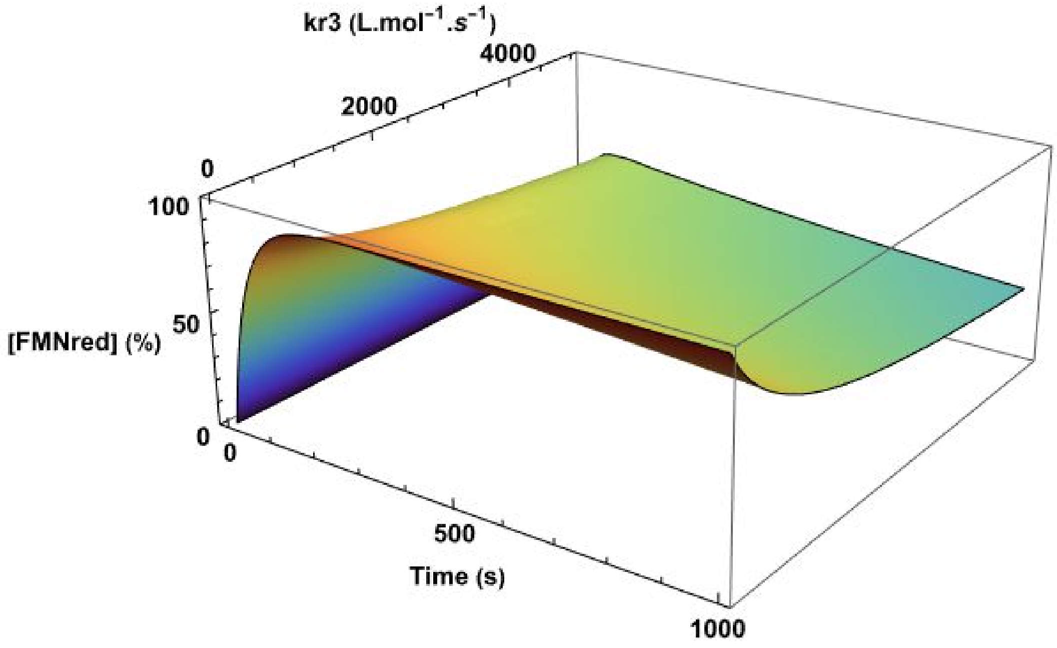

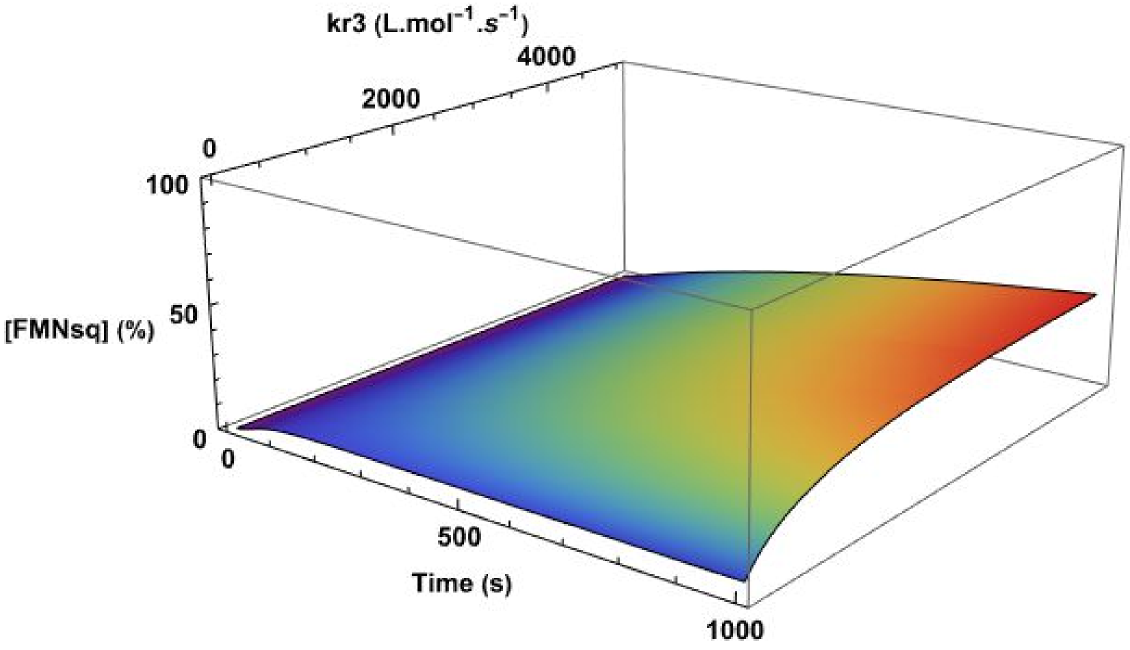

Figure S5: Global numerical analysis of the absorbance variation over time at 19 wavelengths from 340 to 520 nm at intervals of 10 nm, upon hGOX reduction by 3.9 mM **DL-F3Lac**. Global fit of the data according to **(A)** mechanism A assuming step 2 is irreversible, **(B)** mechanism A assuming step 2 is reversible and **(C)** mechanism IntraA assuming an electron transfer between subunits of the same enzyme tetramer. Dotted lines represent the experimental data whereas solid lines correspond to the best fit. The residuals associated with each fit are shown below. The AIC values clearly highlight that the quality of the fit is dramatically improved by implementing reversibility for step 2 in mechanism A, in line with the large improvement of the residuals. Furthermore, inspection of the residuals resulting from the adjustment of the data with mechanism IntraA suggests that the fit is of poorer quality than with mechanism A (step 2 reversible). This is corroborated by the values of the AIC criterion which are higher with mechanism IntraA than those obtained with mechanism A. Taking into account the reversibility of steps 2 and/or 3 for mechanism IntraA does not improve the quality of the fit. Indeed, the AIC values are even higher (data not shown).

The SSQ and AIC values estimated for the kinetic models as explained in Materials and Methods are summarized in the following table, where p is the number of parameters to be fitted:

Mechanism		SSQ	p	N	AIC
A	Step 2: irreversible	0.4281	59	11400	-116042
	Step 2: reversible	0.0065	60	11400	-163719
IntraA	Step 2: irreversible	0.0085	59	11400	-160661

A Mechanism A: step 2 is irreversible

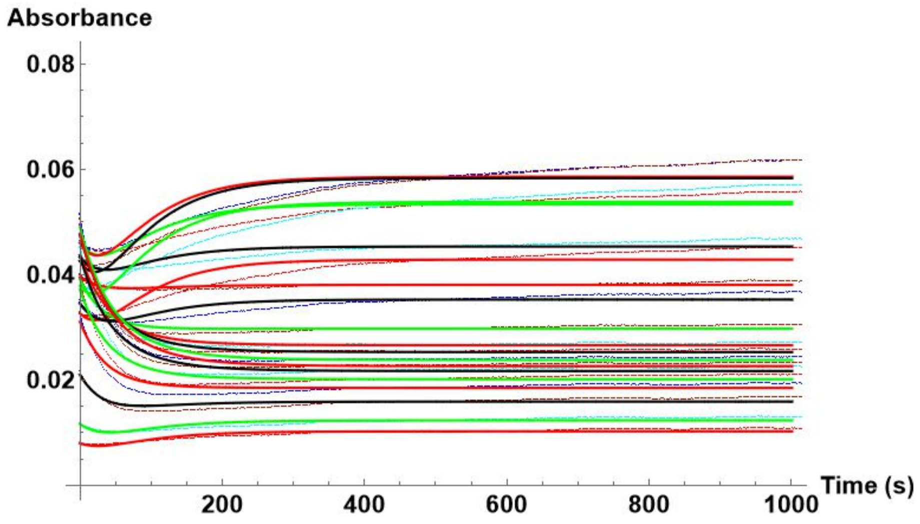

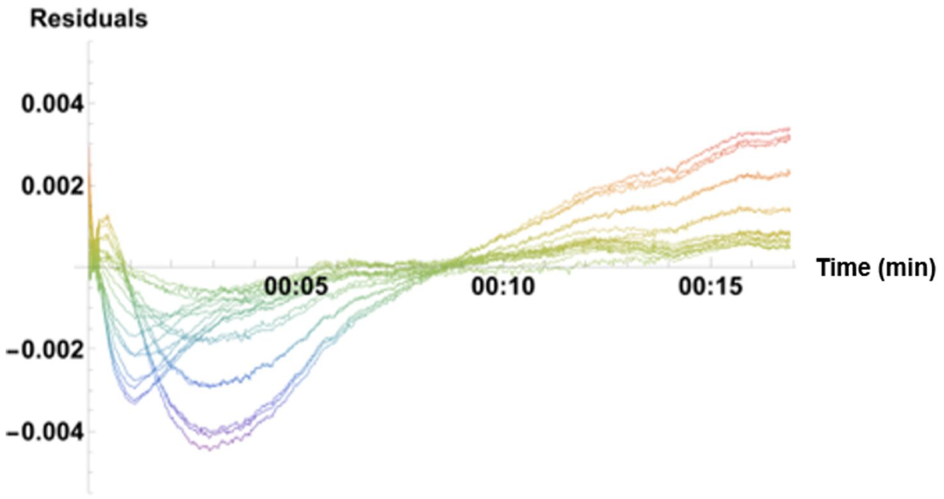

B Mechanism A: step 2 is reversible

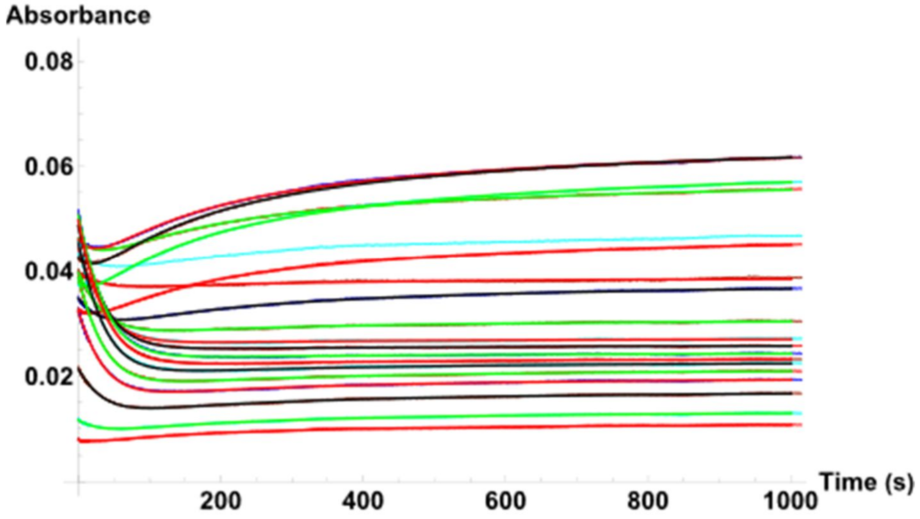

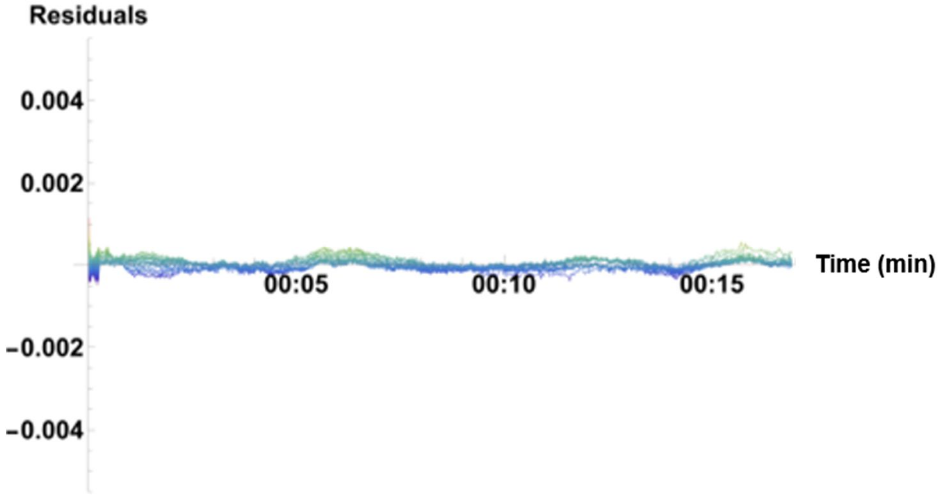

C Mechanism IntraA

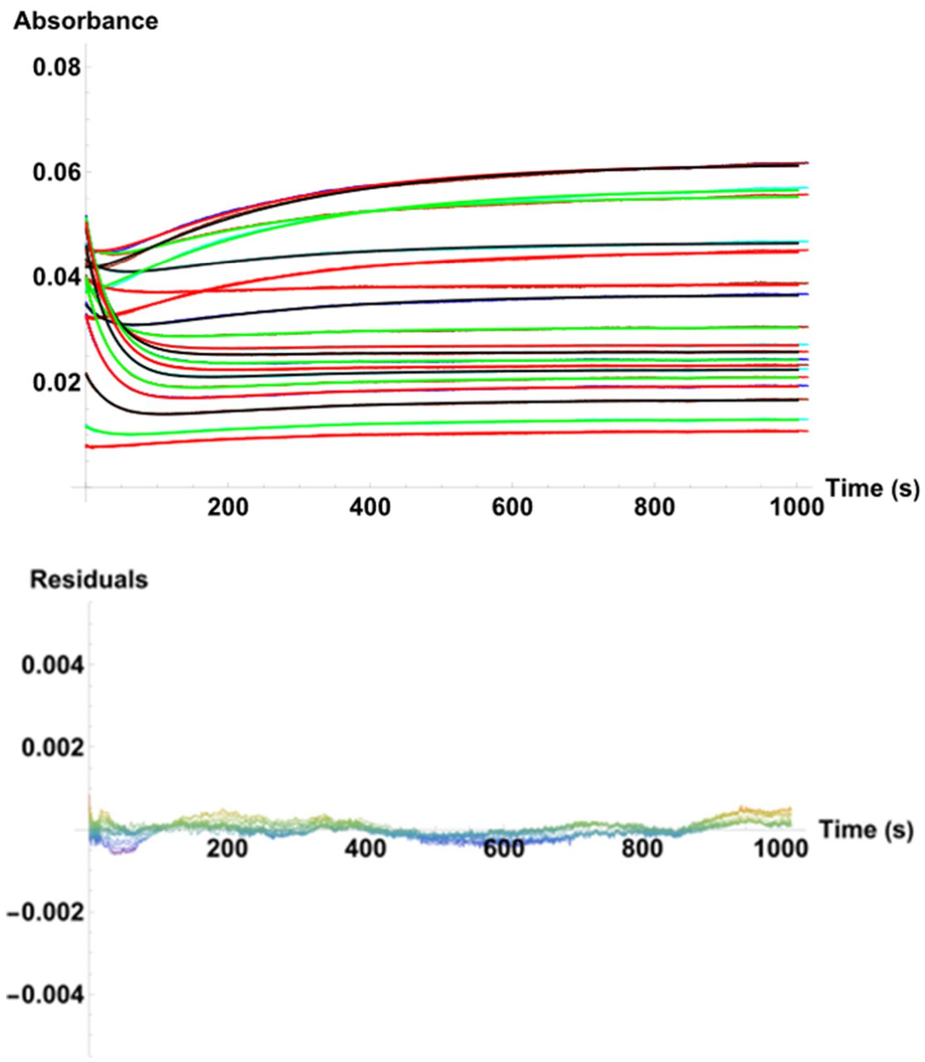

Figure S6: Global numerical analysis of the absorbance variation over time at 19 wavelengths from 340 to 520 nm, at intervals of 10 nm upon hGOX reduction by 10 mM **L-Lactate**. Global fit of the data according to **(A)** mechanism A assuming step 2 is reversible, and **(B)** mechanism IntraA. The enzyme concentration was in this case of the order of 3 μM. While the residuals obtained for mechanism A are satisfactory, those obtained for mechanism IntraA show strong variations. These observations are in agreement with a lower value of the AIC criterion for mechanism A, suggesting once again a better fit of the data with this inter-tetramer mechanism (mechanism A). In addition, the introduction of reversibility for step 2 in mechanism IntraA does not improve the quality of the fit. Dotted lines represent the experimental data whereas solid lines correspond to the best fit. The residuals associated with each fit are shown below. The SSQ and AIC values estimated for all kinetic models as explained in Materials and Methods are summarized in the following table, where p is the number of parameters to be fitted:

	SSQ	p	N	AIC
Mechanism A	0.026	60	4749	-57496
Mechanism IntraA	1.063	60	4749	-39792

A Mechanism A: step 2 is reversible

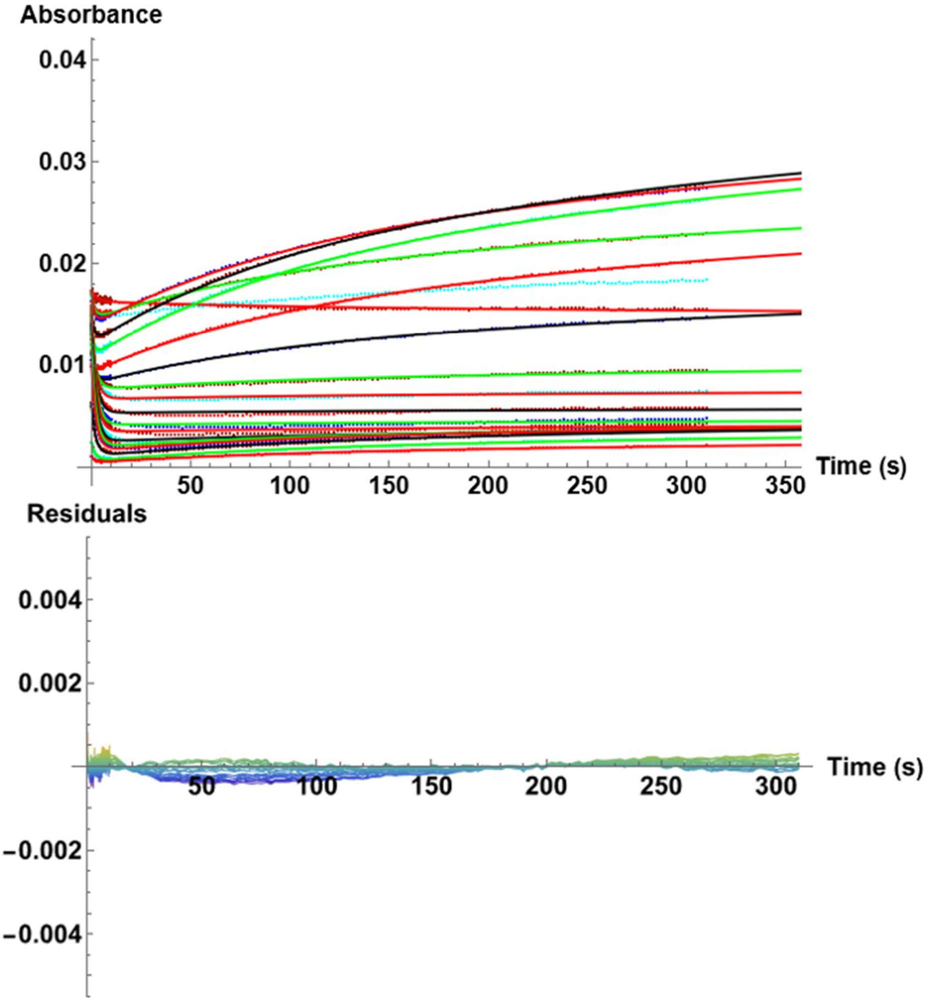

B Mechanism IntraA

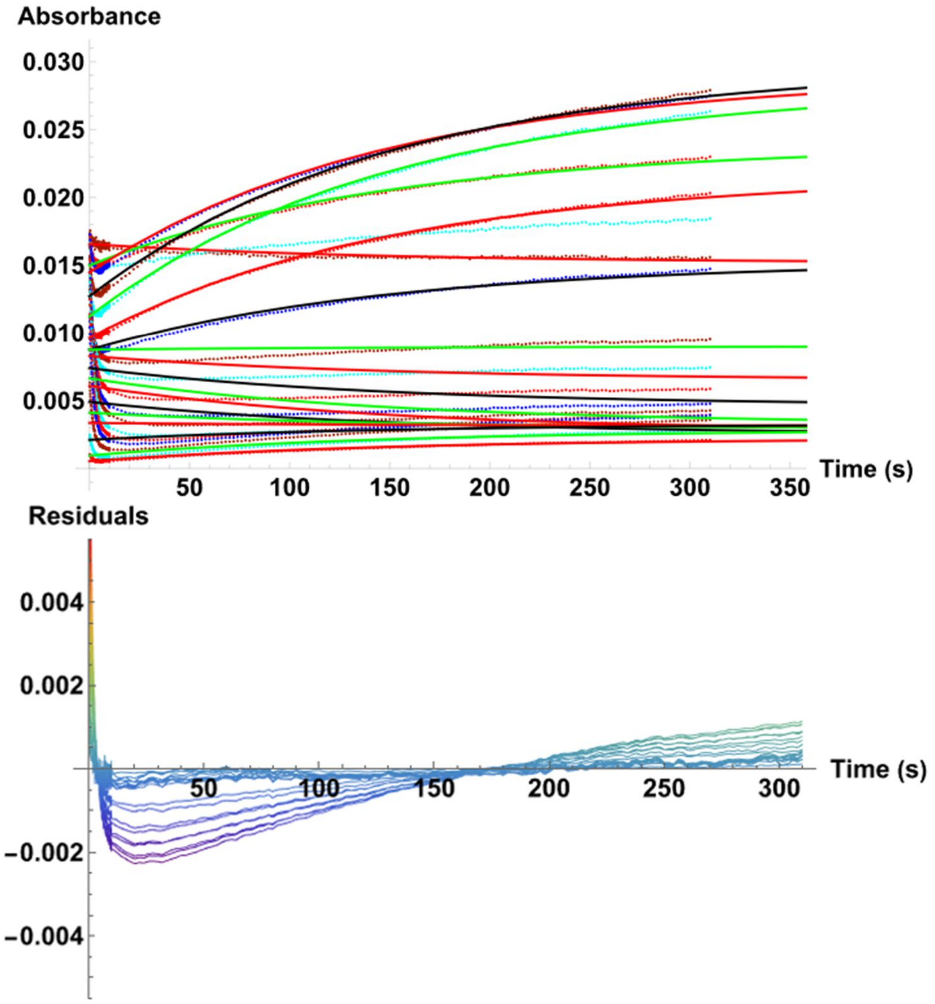

Figure S7: Global numerical analysis of the absorbance variation over time at 19 wavelengths from 340 to 520 nm, at intervals of 10 nm upon hGOX reduction by 5 mM **glycolate**. Global fit of the data according to **(A)** mechanism A assuming step 2 is reversible, **(B)** mechanism B, **(C)** mechanism IntraA and **(D)** mechanism IntraB. Dotted lines represent the experimental data whereas solid lines correspond to the best fit. The residuals associated with each fit are shown below. Whatever the glycolate concentration, when datasets are fitted according to mechanism A, the residuals show a curved pattern in contrast to the residuals from the fit with mechanism B that show no specific pattern. This improvement is corroborated by the value of the AIC criterion which was decreased following the introduction of the intermediate complex ErSo in mechanism B. The mechanism IntraA leads to both a large AIC and residuals showing a strongly curved pattern. With the mechanism IntraB, the addition of an intermediate complex formation, as for mechanism B, improves the fit of the data. However, compared to the fits obtained with the inter-tetramers mechanism B, the values of the AIC criterion are slightly higher and the residuals retain a curved pattern indicating a poorer fit with mechanism IntraB than with mechanism B. The SSQ and AIC values estimated for all kinetic models as explained in Materials and Methods are summarized in the following table, where p is the number of parameters to be fitted:

	SSQ	p	N	AIC
Mechanism A	0.374	60	19000	-205762
Mechanism B	0.209	80	19000	-216807
Mechanism IntraA	0.517	60	19000	-199588
Mechanism IntraB	0.239	81	19000	-214207

A Mechanism A: step 2 is reversible

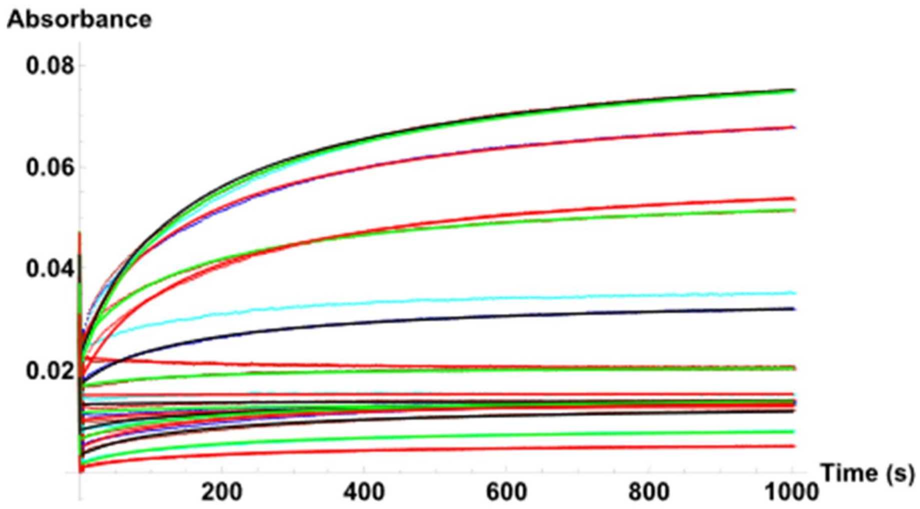

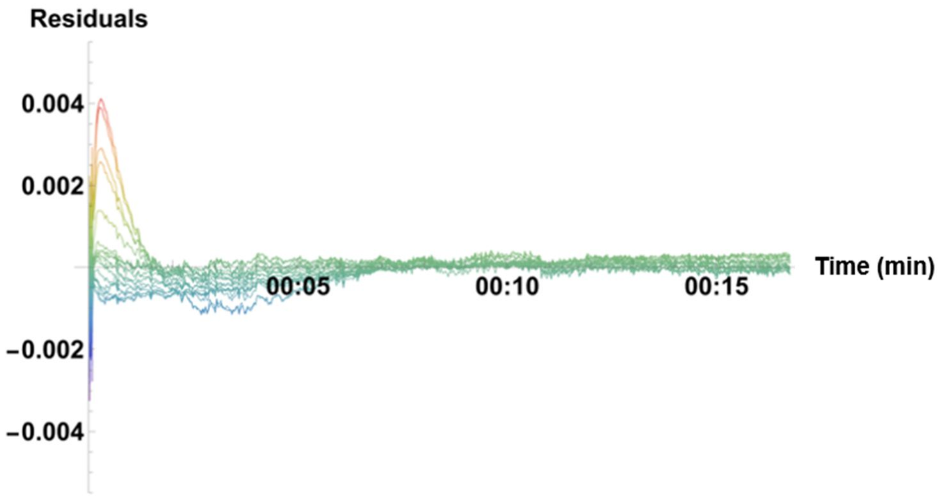

B Mechanism B

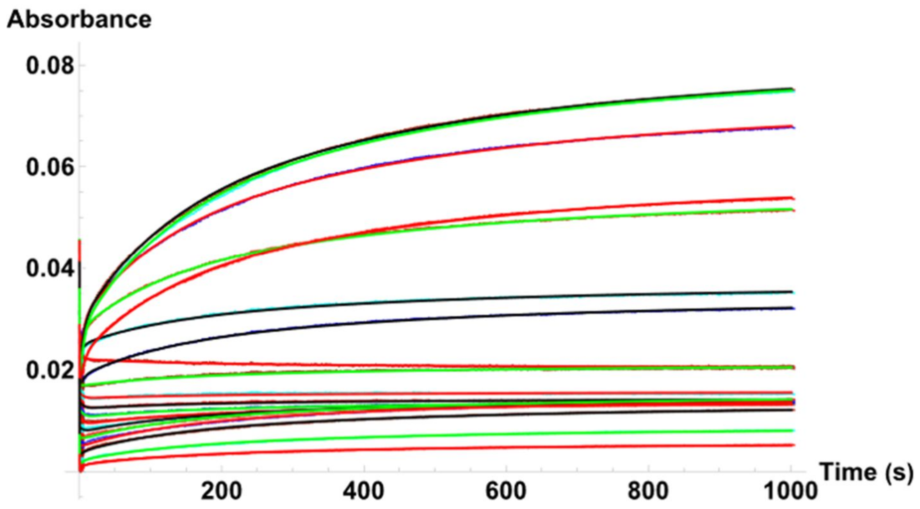

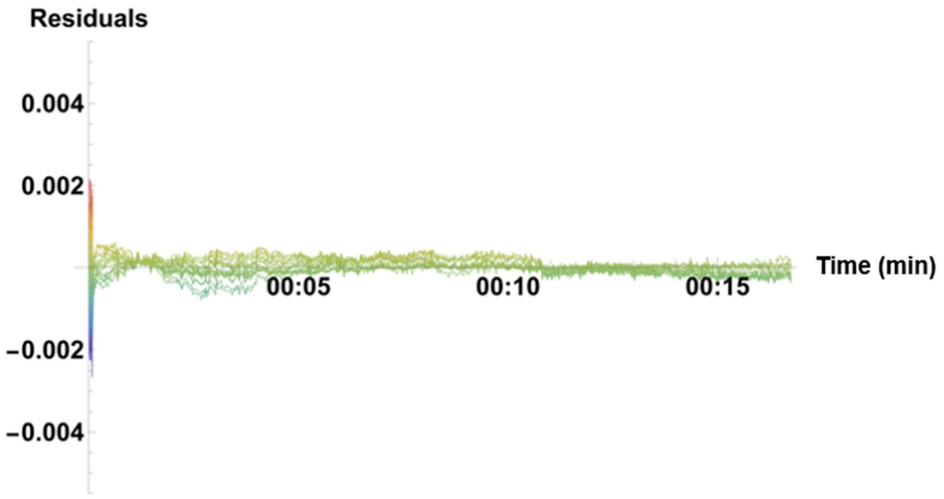

C Mechanism IntraA

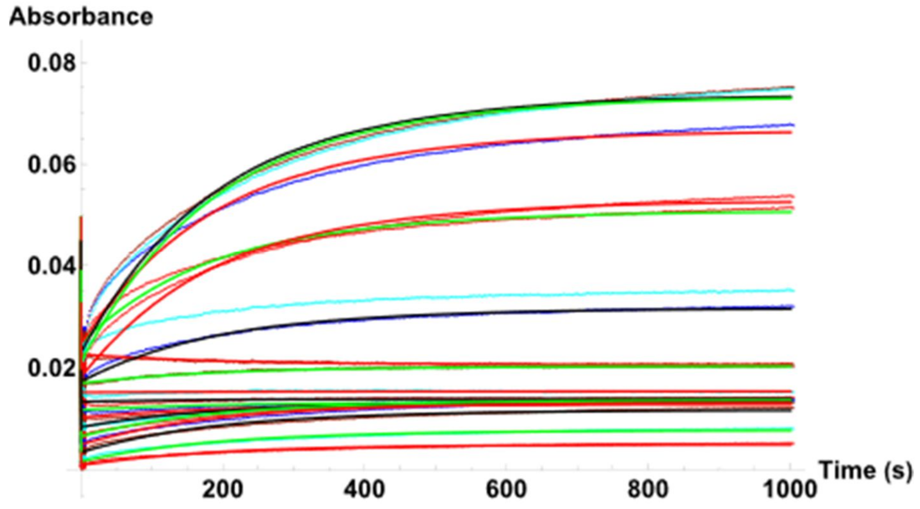

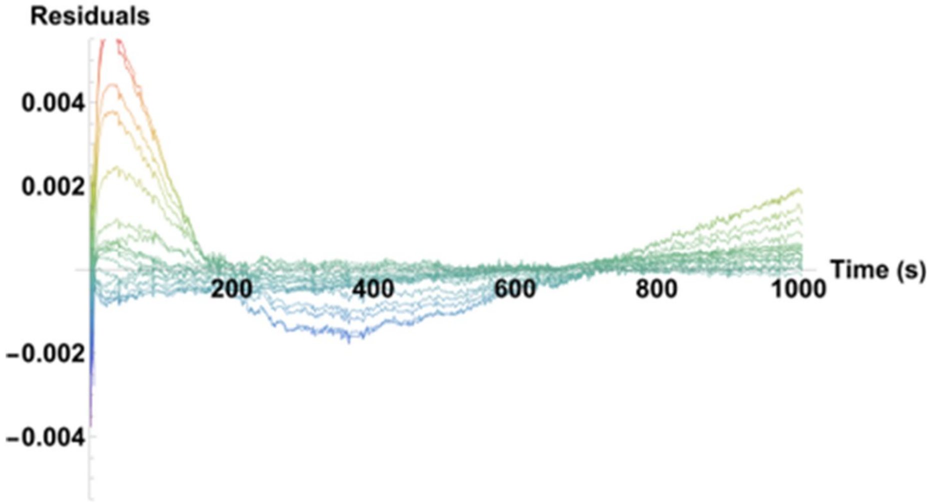

D Mechanism IntraB

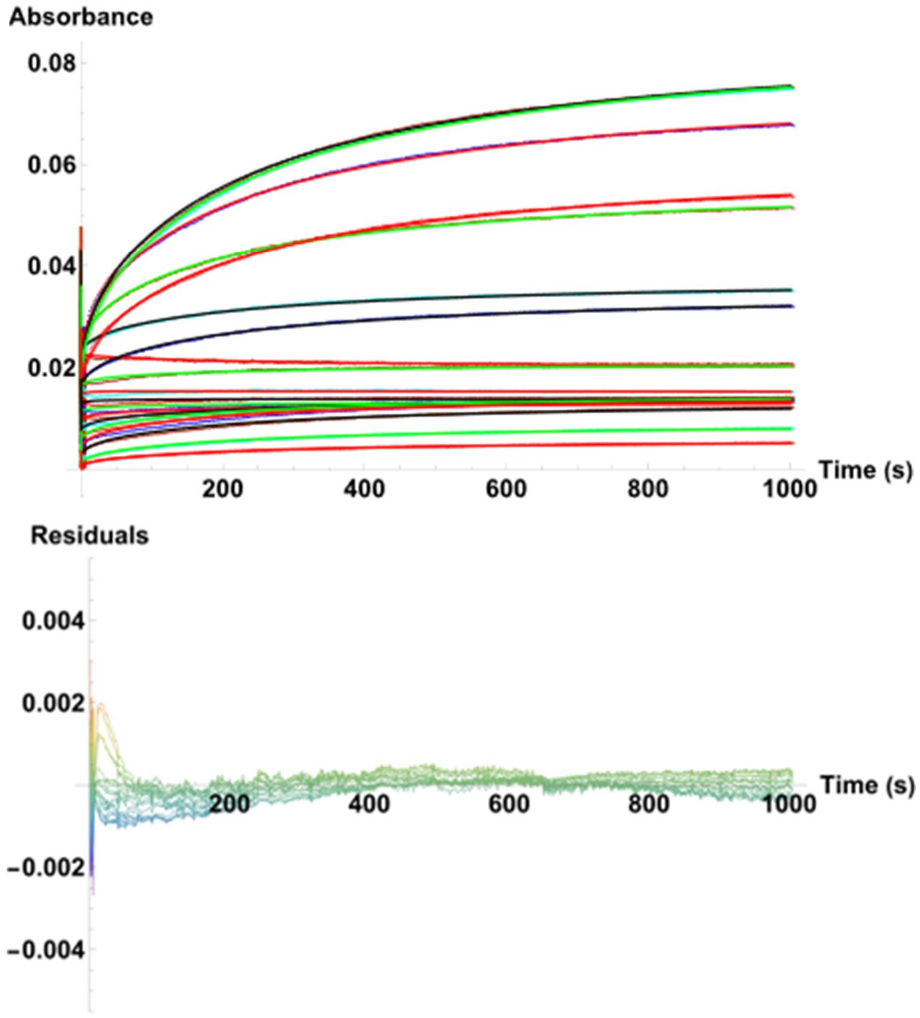

Figure S8. Dependence of the kinetic parameters on **glycolate** concentration. Error bars represent the standard deviations (SD).

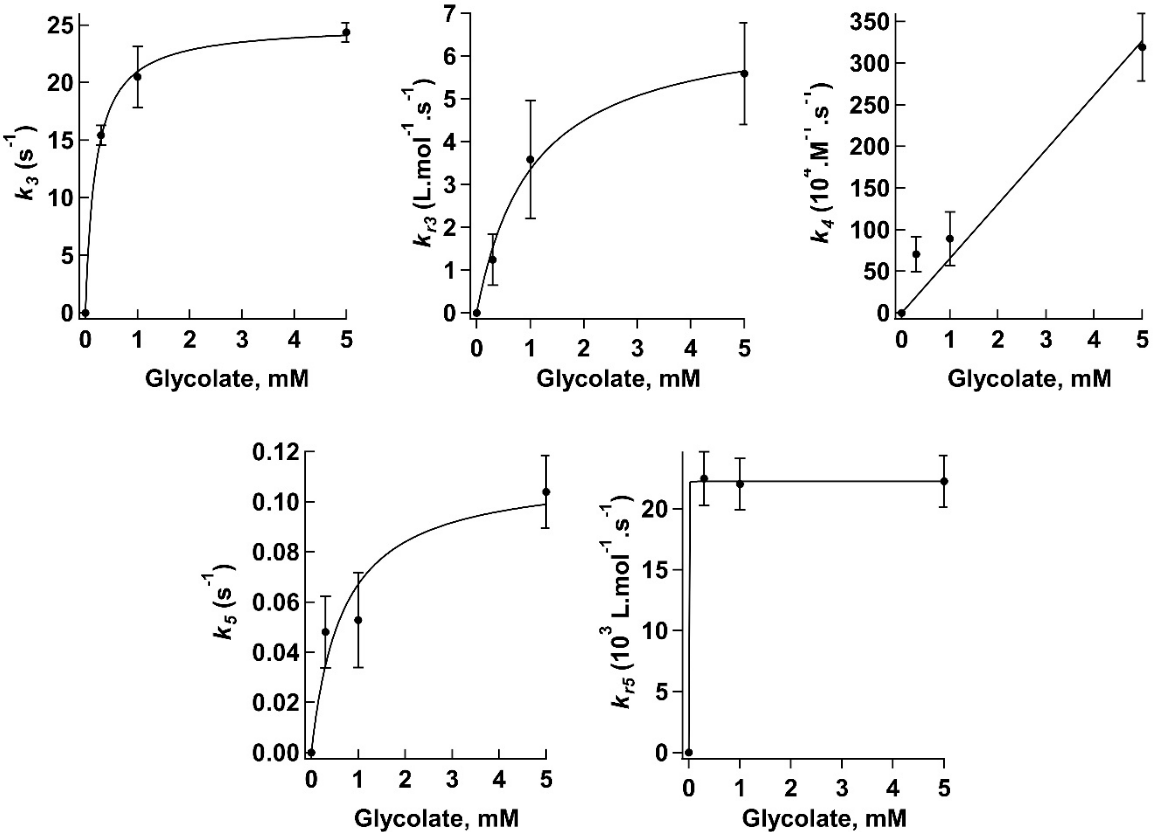

Figure S9. Dependence of the kinetic parameters on **lactate** concentration. Error bars represent the standard deviations (SD).

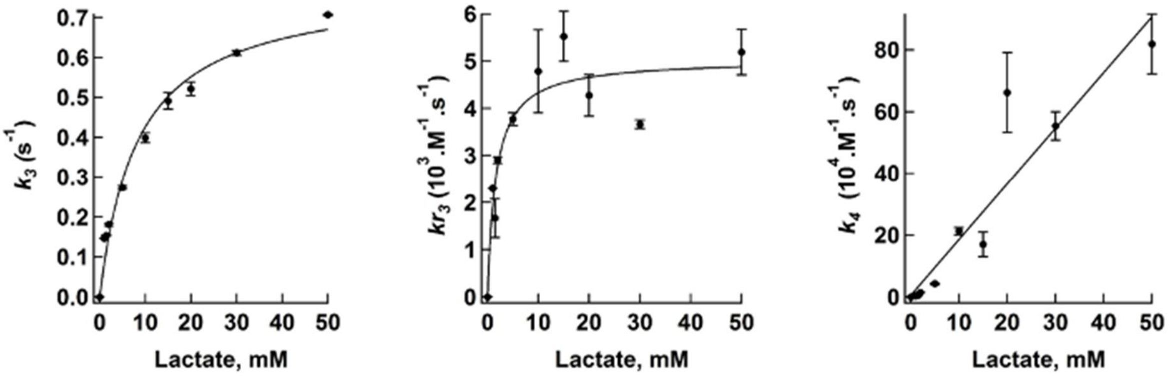

Figure S10. Reaction with glycolate: spectra of the three redox states resulting from the global analysis of the experimental data. Error bars represent the standard deviations (SD).

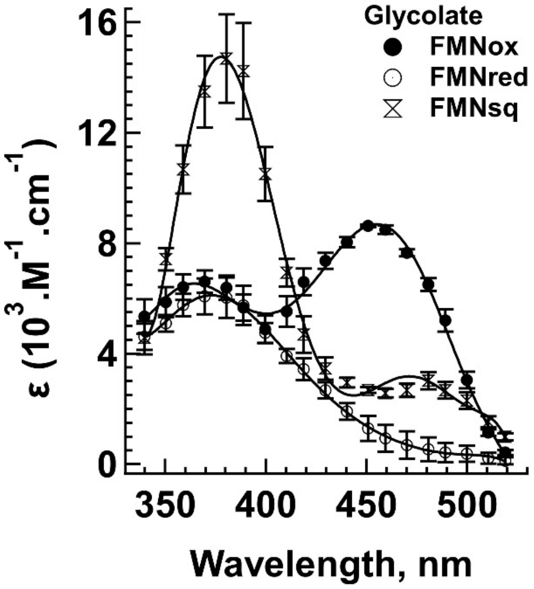

Figure S11. Reaction with lactate: spectra of the three redox states resulting from the global analysis of the experimental data. Error bars represent the standard deviations (SD).

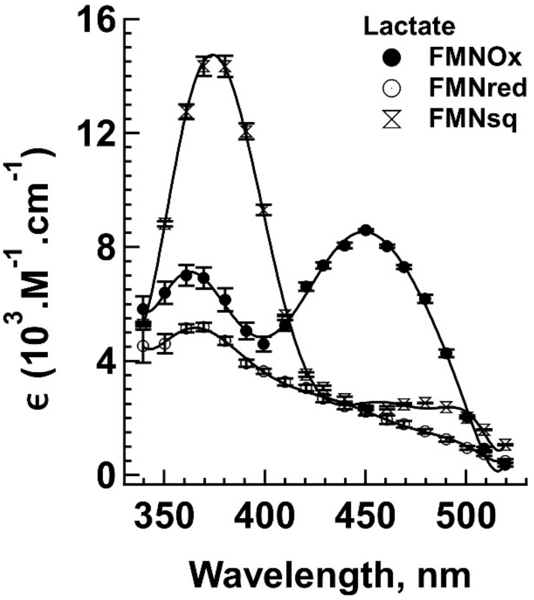

Global fit analysis and equations. In the present work, several mechanisms have been proposed to interpret the formation of the flavin semiquinone form following substrate oxidation by the enzyme. Mechanisms A and B assume a one electron transfer from a reduced flavin to an oxidized one in two separate enzyme tetramers. These two mechanisms A and B are shown below (reproduced from the Results section). Two additional mechanisms (IntraA and IntraB), assuming an intramolecular electron transfer between subunits of an enzyme tetramer were also considered; as explained in the text, these mechanisms are not satisfactory. These two intramolecular mechanisms are described below and some examples of fits are shown in Figure S5 (F3Lac), Figure S6 (Lactate) and Figure S7 (Glycolate).

Mechanism A

$$\underset{\textbf{Eox}}{\text{FMNox}} + \underset{\textbf{Sred}}{\text{Sred}} \underset{k_2}{\overset{k_1}{\rightleftharpoons}} \underset{\textbf{EoSr}}{\text{FMNox-Sred}} \underset{k_{r3}}{\overset{k_3}{\rightleftharpoons}} \underset{\textbf{Ered}}{\text{FMNred}} + \text{Sox}$$

Step 1 (k1/k2); *Step 2* (k3/kr3)

$$\underset{\textbf{Eox}}{\text{FMNox}} + \underset{\textbf{Ered}}{\text{FMNred}} \xrightarrow{k_4} \underset{\textbf{Esq}}{2\ \text{FMNsq}}$$

Step 3

Mechanism B

$$\underset{\textbf{Eox}}{\text{FMNox}} + \underset{\textbf{Sred}}{\text{Sred}} \underset{k_2}{\overset{k_1}{\rightleftharpoons}} \underset{\textbf{EoSr}}{\text{FMNox-Sred}} \underset{k_{r3}}{\overset{k_3}{\rightleftharpoons}} \underset{\textbf{ErSo}}{\text{FMNred-Sox}} \underset{k_{r5}}{\overset{k_5}{\rightleftharpoons}} \underset{\textbf{Ered}}{\text{FMNred}} + \text{Sox}$$

Step 1 (k1/k2); *Step 2* (k3/kr3); *Step 3* (k5/kr5)

$$\underset{\textbf{Eox}}{\text{FMNox}} + \underset{\textbf{Ered}}{\text{FMNred}} \xrightarrow{k_4} \underset{\textbf{Esq}}{2\ \text{FMNsq}}$$

Step 4

Mechanism IntraA

$$\underset{\textbf{Eox}}{\text{FMNox,ox}} + \underset{\textbf{Sred}}{\text{Sred}} \underset{k_2}{\overset{k_1}{\rightleftharpoons}} \underset{\textbf{EoSr}}{\text{FMNox,ox-Sred}} \underset{k_{r3}}{\overset{k_3}{\rightleftharpoons}} \underset{\textbf{Ered}}{\text{FMNox,red}} + \text{Sox}$$

Step 1 (k1/k2); *Step 2* (k3/kr3)

$$\underset{\textbf{Ered}}{\text{FMNox,red}} \xrightarrow{k_4} \underset{\textbf{Esq}}{\text{FMNsq,sq}}$$

Step 3

Mechanism IntraB

$$\underset{\textbf{Eox}}{\text{FMNox,ox}} + \underset{\textbf{Sred}}{\text{Sred}} \underset{k_2}{\overset{k_1}{\rightleftharpoons}} \underset{\textbf{EoSr}}{\text{FMNox,ox-Sred}} \underset{k_{r3}}{\overset{k_3}{\rightleftharpoons}} \underset{\textbf{ErSo}}{\text{FMNox,red-Sox}} \underset{k_{r5}}{\overset{k_5}{\rightleftharpoons}} \underset{\textbf{Ered}}{\text{FMNox,red}} + \text{Sox}$$

Step 1 (k1/k2); *Step 2* (k3/kr3); *Step 3* (k5/kr5)

$$\underset{\textbf{Ered}}{\text{FMNox,red}} \xrightarrow{k_4} \underset{\textbf{Esq}}{\text{FMNsq,sq}}$$

Step 4

For all mechanisms, the differential equations describing the rate of change of the concentration of all chemical species with time are presented below:

Mechanism A

$$\frac{d[Eox](t)}{dt} = -\mathrm{k1}.[Eox](t).[Sred](t) + \mathrm{k2}.[EoSr](t) - \mathrm{k4}.[Eox](t).[Ered](t)$$
$$\frac{d[Sred](t)}{dt} = -\mathrm{k1}.[Eox](t).[Sred](t) + \mathrm{k2}.[EoSr](t)$$
$$\frac{d[EoSr](t)}{dt} = \mathrm{k1}.[Eox](t).[Sred](t) - (\mathrm{k2} + \mathrm{k3}).[EoSr](t) + \mathrm{kr3}.[Ered](t).[Sox](t)$$
$$\frac{d[Ered](t)}{dt} = \mathrm{k3}.[EoSr](t) - \mathrm{kr3}.[Ered](t).[Sox](t) - \mathrm{k4}.[Eox](t).[Ered](t)$$
$$\frac{d[Sox](t)}{dt} = \mathrm{k3}.[EoSr](t) - \mathrm{kr3}.[Ered](t).[Sox](t)$$
$$\frac{d[Esq](t)}{dt} = 2.\mathrm{k4}.[Eox](t).[Ered](t)$$

Mechanism B :

$$\frac{d[Eox](t)}{dt} = -\mathrm{k1}.[Eox](t).[Sred](t) + \mathrm{k2}.[EoSr](t) - \mathrm{k4}.[Eox](t).[Ered](t)$$
$$\frac{d[Sred](t)}{dt} = -\mathrm{k1}.[Eox](t).[Sred](t) + \mathrm{k2}.[EoSr](t)$$
$$\frac{d[EoSr](t)}{dt} = \mathrm{k1}.[Eox](t).[Sred](t) - (\mathrm{k2} + \mathrm{k3}).[EoSr](t) + \mathrm{kr3}.[ErSo](t)$$
$$\frac{d[ErSo](t)}{dt} = k3.[EoSr](t) - (\mathrm{kr3} + \mathrm{k5}).[ErSo](t) + kr5.[Ered](t).[Sox](t)$$
$$\frac{d[Ered](t)}{dt} = \mathrm{k5}.[ErSo](t) - \mathrm{kr5}.[Ered](t).[Sox](t) - \mathrm{k4}.[Eox](t).[Ered](t)$$
$$\frac{d[Sox](t)}{dt} = \mathrm{k5}.[ErSo](t) - \mathrm{kr5}.[Ered](t).[Sox](t)$$
$$\frac{d[Esq](t)}{dt} = 2.k4.[Eox](t).[Ered](t)$$

Mechanism IntraA

$$\frac{d[Eox](t)}{dt} = -\mathrm{k1}.[Eox](t).[Sred](t) + \mathrm{k2}.[EoSr](t)$$
$$\frac{d[Sred](t)}{dt} = -\mathrm{k1}.[Eox](t).[Sred](t) + \mathrm{k2}.[EoSr](t)$$
$$\frac{d[EoSr](t)}{dt} = \mathrm{k1}.[Eox](t).[Sred](t) - (\mathrm{k2} + \mathrm{k3}).[EoSr](t) + \mathrm{kr3}.[Ered](t).[Sox](t)$$
$$\frac{d[Ered](t)}{dt} = \mathrm{k3}.[EoSr](t) - \mathrm{kr3}.[Ered](t).[Sox](t) - \mathrm{k4}.[Ered](t)$$
$$\frac{d[Sox](t)}{dt} = \mathrm{k3}.[EoSr](t) - \mathrm{kr3}.[Ered](t).[Sox](t)$$
$$\frac{d[Esq](t)}{dt} = \mathrm{k4}.[Ered](t)$$

Mechanism IntraB :

$$\frac{d[Eox](t)}{dt} = -\mathrm{k1}.[Eox](t).[Sred](t) + \mathrm{k2}.[EoSr](t)$$

$$\frac{d[Sred](t)}{dt} = -\mathrm{k1}.[Eox](t).[Sred](t) + \mathrm{k2}.[EoSr](t)$$

$$\frac{d[EoSr](t)}{dt} = \mathrm{k1}.[Eox](t).[Sred](t) - (\mathrm{k2} + \mathrm{k3}).[EoSr](t) + \mathrm{kr3}.[ErSo](t)$$

$$\frac{d[ErSo](t)}{dt} = k3.[EoSr](t) - (\mathrm{kr3} + \mathrm{k5}).[ErSo](t) + kr5.[Ered](t).[Sox](t)$$

$$\frac{d[Ered](t)}{dt} = \mathrm{k5}.[ErSo](t) - \mathrm{kr5}.[Ered](t).[Sox](t) - \mathrm{k4}.[Ered](t)$$

$$\frac{d[Sox](t)}{dt} = \mathrm{k5}.[ErSo](t) - \mathrm{kr5}.[Ered](t).[Sox](t)$$

$$\frac{d[Esq](t)}{dt} = \mathrm{k4}.[Ered](t)$$

For all mechanisms, the initial conditions were given as [Eox, Sred, EoSr, Ered, Sox, Esq](0)=[E_0,S_0, 0, 0, 0, 0] mol.L^{-1}. For mechanisms B and intraB, the initial concentration of the intermediate complex ErSo was also set to 0 mol.L^{-1}. For each fitted parameter, the p-value was calculated. This parameter indicates whether or not the estimated value of the parameters is significantly different from 0. With mechanism A (with reversible step 2), the p-value for all fitting parameters is null, which means that these parameters contribute significantly to the model. With mechanism B, all the parameters were found associated with a value of zero for the p-value, except for the molar extinction coefficients of the intermediate complex ErSo for the wavelengths from 430 nm to 520 nm. This may be attributable to the low amplitude of the absorbance signal at these wavelengths, in the time range where this species contributes to the signal i.e. a few seconds. It is likely that the data do not contain enough information to allow an accurate estimate of these coefficients in this wavelength range.

The p-values for all parameters are also found to be zero in the case of mechanism IntraA, under the assumption that step 2 is irreversible. This is no longer the case when step 2 is considered reversible for which the p-value of the kr3 parameter is non-zero. This is not surprising, since the fully oxidized form FMNox,ox plays no role in the semiquinone formation. A rather similar behaviour is observed for mechanism IntraB. When step 2 is considered as reversible, the p-value of the kr3 parameter is non-zero. Moreover, as for mechanism B, the molar extinction coefficients of the intermediate complex ErSo for the wavelengths from 430 nm to 520 nm are characterized by non-zero p-values. In addition, the molar extinction coefficients of the first intermediate complex EoSr for the wavelengths from 420 nm to 500 nm are also characterized by non-zero p-values.

Scheme S1: Principle of the transhydrogenation reaction.

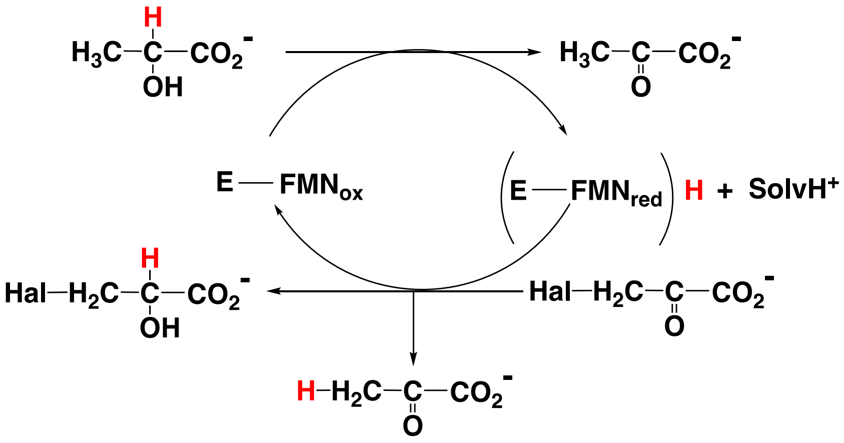

The enzyme is incubated in the presence of a saturating concentration of lactate (deuterated or not at the C2 position) and varying concentrations of a halogeno-ketoacid. The reducing substrate α-hydrogen or isotope (in red) is taken either by the flavin (hydride transfer mechanism) or by the catalytic histidine (carbanion mechanism). It has been shown to be sticky and is transferred in part to the products of the oxidizing reverse substrate as shown above. If the halogen is eliminated by attack of a hydride from the reduced flavin N5H, a normal isotope effect is expected for the dehalogenation reaction. When the reaction goes *via* a carbanion, the latter partitions between an isotope-sensitive route (protonation from the histidine N3H) and an isotope insensitive route, namely an intramolecular halogen elimination (the intermediate enolate can pick up some isotope from the solvent). Halogen elimination is thus accelerated and one can expect an inverse deuterium isotope effect for that route. Experimentally, with the wild type enzyme and a few variant ones, a normal isotope effect was found for overall disappearance of bromopyruvate, but an inverse one for the dehalogenation reaction (Urban *et al*. (1985) J. Biol. Chem., 260, 11115-11122; Dubois, J. et al.(1990) Biochemistry *29*, 6393-6400; Bodevin, S. and Lederer, F., In *Flavins and Flavoproteins 1999*, Ghisla, S., Kroneck, P., Macheroux, P., Sund, H., Ed. Rudolf Weber, 1999, pp 463-466).

Codes of PDB (Protein data Bank) crystal structures of ternary complexes of alcohol dehydrogenases with NAD^+ and trifluoroethanol **:**

- **from horse liver:** 1A71, 1AXE, 1AXG, 4DXH, 4NFS, 5KCZ, 5KJF, 6OA7, 6OWP
- **from yeast:** 4W6Z, 5ENV, 7KCB
